# Anthropogenic impacts on threatened species erode functional diversity in chelonians and crocodilians

**DOI:** 10.1038/s41467-023-37089-5

**Published:** 2023-03-28

**Authors:** R. C. Rodríguez-Caro, E. Graciá, S. P. Blomberg, H. Cayuela, M. Grace, C. P. Carmona, H. A. Pérez-Mendoza, A. Giménez, R. Salguero-Gómez

**Affiliations:** 1 Department of Biology, University of Oxford, 11a Mansfield Road, Oxford, OX1 3SZ UK; 2 Departamento de Biología Aplicada, Universidad Miguel Hernández, Elche, 03202 Alicante, Spain; 3 Departamento de Ecología, Universidad de Alicante, San Vicent del Raspeig, 03690 Alicante, Spain; 4 Centro de Investigación e Innovación Agroalimentaria y Agroambiental (CIAGRO-UMH), Universidad Miguel Hernández, 03312 Orihuela, Spain; 5 School of Biological Sciences, The University of Queensland, Brisbane, QLD 4072 Australia; 6 Laboratoire de Biométrie et Biologie Evolutive, Université Lyon 1, CNRS, UMR 5558, F-769622 Villeurbanne, France; 7 Institute of Ecology and Earth Sciences, University of Tartu, 50409 Tartu, Estonia; 8 Facultad de Estudios Superiores Iztacala, Universidad Autónoma de México, 54090 Tlalnepantla, México; 9 Max Plank Institute for Demographic Research, Konrad-Zuße Straße 1, 18057 Rostock, Germany

**Keywords:** Conservation biology, Herpetology, Macroecology

## Abstract

The Anthropocene is tightly associated with a drastic loss of species worldwide and the disappearance of their key ecosystem functions. The orders Testudines (turtles and tortoises) and Crocodilia (crocodiles, alligators, and gharials) contain numerous threatened, long-lived species for which the functional diversity and potential erosion by anthropogenic impacts remains unknown. Here, we examine 259 (69%) of the existing 375 species of Testudines and Crocodilia, quantifying their life history strategies (i.e., trade-offs in survival, development, and reproduction) from open-access data on demography, ancestry, and threats. We find that the loss of functional diversity in simulated extinction scenarios of threatened species is greater than expected by chance. Moreover, the effects of unsustainable local consumption, diseases, and pollution are associated with life history strategies. In contrast, climate change, habitat disturbance, and global trade affect species independent of their life history strategy. Importantly, the loss of functional diversity for threatened species by habitat degradation is twice that for all other threats. Our findings highlight the importance of conservation programmes focused on preserving the functional diversity of life history strategies jointly with the phylogenetic representativity of these highly threatened groups.

## Abstract (web summary)

Many species of reptiles are threatened with extinction. This analysis on 259 species of turtles, tortoises and crocodilians identifies anthropogenic threats that disproportionally affect species with certain life history strategies and others that affect all species similarly.

## Introduction

Human impacts, such as habitat loss, climate change, pollution, poaching, and unsustainable trade, impose major threats to the persistence of species worldwide^1^. Indeed, modern human activities have drastically accelerated species extinctions by three orders of magnitude over pre-human extinctions levels^2^. As such, effective conservation measures are urgently needed to bend the curve of biodiversity loss^3^.

On-going biodiversity loss is resulting in the erosion of functional diversity, key to ecological function^4–6^. A robust way to characterise functional diversity across a clade is via quantifying life history strategies, i.e. trade-offs between an individual’s investment in survival, development, and reproduction driven by limited resources and physiological constraints^7–11^. In this context, different species can possess similar life history trait values, resulting in functional redundancy^12^ (e.g., the Texas tortoises, *Gopherus berlandieri*, and the common snapping turtle, *Chelydra serpentina*, achieve similar longevities^13^), or display dissimilar functionality, giving rise to functional dispersion^14^ (e.g., the loggerhead sea turtle, *Caretta caretta*, reproduces later than other Testudines of similar size, such as the Aldabra giant tortoise, *Aldabrachelys gigantea*^15^).

Evaluating the risk of extinction of long-lived species is important for an accurate understanding and preservation of their functional diversity. Critically, the extinction of long-lived species can be delayed due to their long generation times^8,11,16,17^. Thus, environmental effects typically manifest at much longer time scales in long-lived species than in short-lived ones^18,19^. Perhaps not surprisingly, two of the most threatened and long-lived groups are the order Testudines (tortoises, turtles, and sea turtles) and Crocodilia (crocodiles, alligators, and gharials)^20–22^. In fact, Testudines and Crocodilia contain some of the highest proportions of threatened species across vertebrates (58% and 50%, respectively^22,23^). Moreover, the actual conservation status of Testudines and Crocodilia is likely worse than IUCN classifications because some Data Deficient species could in fact be threatened^24^. Indeed, 25% of the Testudines and Crocodilia lack reliable data to accurately identify their IUCN status^20^, but a recent study across all reptiles suggests that 59% of 1032 Data Deficient species could be threatened by extinction^24^.

Here, we characterise the life history strategies of 69% of Testudines and Crocodilia species—259 out of ~375 extant species—to identify how the impacts of different human-led global (climate change, global wildlife trade, and interactions with invasive species and diseases) and local threats (habitat degradation, unsustainable local consumption, and pollution) may alter their functional diversity. Using demographic and phylogenetic open-access data, we quantify life history strategies to estimate their functional diversity. We then simulate different extinction scenarios according to their extinction risk and the main threats that affect each species. We test the following hypotheses: (H1) the loss of functional diversity due to the extinction of threatened species will be lower than expected by chance. The reason is that the life history strategies across testudines and crocodilians are expected to show high functional redundancy, since most of them are long-lived species with delayed maturity^10^. (H2) human threats (e.g., global trade, unsustainable local consumption, interactions with invasive species and diseases) will differentially erode the functional diversity of these species^25^. For instance, some evidence exists that tortoise and turtle populations may be declining due to habitat degradation, unsustainable local consumption, and international pet trade collection^26^. In contrast, threats to the functional diversity of these species due to global climate change may a priori not seem more intense than local threats^22^. Finally, we find that the current scenario of species extinction based on the IUCN threatened status would result in a worst-case scenario for the loss of reptile functional diversity. This finding highlights how threatened reptile species display unique life history strategies.

## Results

### High functional redundancy

**Fig. 1:**
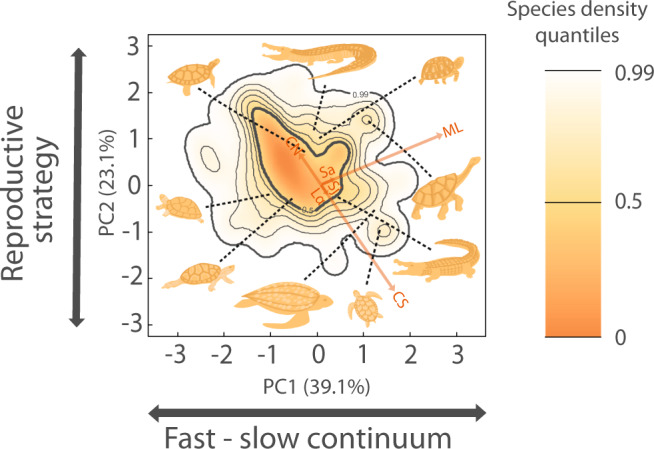
The global functional spectra of the life history strategies of Testudines and Crocodilia is described by two dominant axes of life history variation: (PC1) fast–slow continuum and (PC2) reproductive strategies. The probabilistic species distribution in this space is defined by the two first principal components axes (PC1 = 39.1% and PC2 = 23.1% of variance explained) of a phylogenetically-corrected principal component analysis (*p*PCA), where adult body mass has also been corrected. The phylogenetic signal is Pagel’s *λ* = 0.674 ± 0.030 (SE). The life history traits are: adult survival (*Sa*), juvenile survival (*Sj*), maximum lifespan (*ML*), age at sexual maturity (*Lα*), mean of number of clutches per year (*CN*), clutch size (*CS*). Arrows indicate the direction and weighting of each trait in the *p*PCA. The colour gradient (orange, yellow, and white) depicts the density of species in the defined space, where orange corresponds to more densely populated areas). Thick contour lines indicate the 0.5 and 0.99 quantiles, and thinner ones indicate quantiles 0.6, 0.7, 0.8, and 0.9. The species silhouettes correspond to (starting at the top left and moving clockwise): painted turtle (*Chrysemys picta*), American alligator (*Alligator mississippiensis*), desert tortoise (*Gopherus agassizii*), Hood Island giant tortoise (*Chelonoidis hoodensis*), Orinoco crocodile (*Crocodylus intermedius*), loggerhead sea turtle (*Caretta caretta*), leatherback sea turtle (*Dermochelys coriacea*), northern map turtle (*Graptemys geographica*), and Western Caspian turtle (*Mauremys rivulata*).

Two dominant axes of life history traits describe most of the functional diversity of Testudines and Crocodilia. Using life history trait data from 236 species of turtles and tortoises (67% of the extant species), and 23 species of crocodilians (85%) from multiple sources, we characterise their life history strategies via a phylogenetic principal component analysis (*p*PCA; Supplementary Tables 1 and 2) accounting for adult body mass (Supplementary Table 3). To address gaps in the dataset (see Supplementary Methods. Missing data), we performed a multiple phylogenetic-trait imputation for missing values (see “Methods”). The two dominant axes of life history strategies encompass 62% of the total functional variation (Fig. 1). These axes correspond to (1) the “fast–slow continuum”^7,27–29^, which separates species with short maximum lifespans, like some freshwater turtles (e.g., 4.3 years for the Alabama red-bellied turtle, *Pseudemys alabamensis*), to species with long maximum lifespans such as terrestrial tortoises (e.g. 176 years for the Aldabra giant tortoise, *Aldabrachelys gigantea*); and (2) the “reproductive strategy”^8,11,16,30,31^, defined by a trade-off between clutch frequency vs. reproductive output, with species with high numbers of clutches per year but lower clutch size per clutch at one end (e.g., 1.2 eggs per clutch and three clutches per year, as in the Madagascan flat-tailed tortoise, *Pyxis planicauda*), and extremely fecund species at the other end, having high clutch sizes but few clutches per year (e.g., 91.5 eggs per clutch and one clutch per year, as in the South American River Turtle, *Podocnemis expansa*).

Examining the functional spectra of the life history traits of testudines and crocodilians reveals a high degree of redundancy. We use a trait probability density (TPD) approach considering the two-dimensional functional space (Fig. 1) to identify peaks (areas of high density) and valleys (low density) of species diversity^6,32^. The examined reptile species tend to congregate around a single hotspot with median values of maximum lifespan and high number of clutches per year with lower clutch sizes. This hotspot of life history strategies includes 50% of the 259 species and represents 13.2% of the total spectrum (Fig. 1), in agreement with a recent analysis examining 6567 species of reptiles (12.3% of the spectrum^5^). Freshwater turtles, such as the eastern long-necked turtle, *Chelodina longicollis* (max. lifespan 37 years, 13.9 eggs/clutch, two clutches/year) and crocodiles, such as the mugger crocodile *Crocodylus palustris* (max. lifespan 31.5 years, 28.75 eggs/clutch, two clutches/year), are located in that hotspot of functional diversity. Sea turtles are represented in a valley of functional diversity (bottom right of Fig. 1), which is characterised by species with higher clutch sizes, few clutches per year, and higher maximum lifespan (e.g. *Caretta caretta*: max. lifespan 76 years, 115 eggs/clutch, 1.29 clutches/year). In contrast, terrestrial tortoises are represented at the top right of Fig. 1, with their typically shorter clutch sizes, high clutches per year, and longer maximum lifespans (e.g., the Aldabra giant tortoise *Aldabrachelys gigantea*: max. lifespan 176 years, 13.5 eggs/clutch, two clutches/year).

### Unique strategies may become extinct

**Fig. 2:**
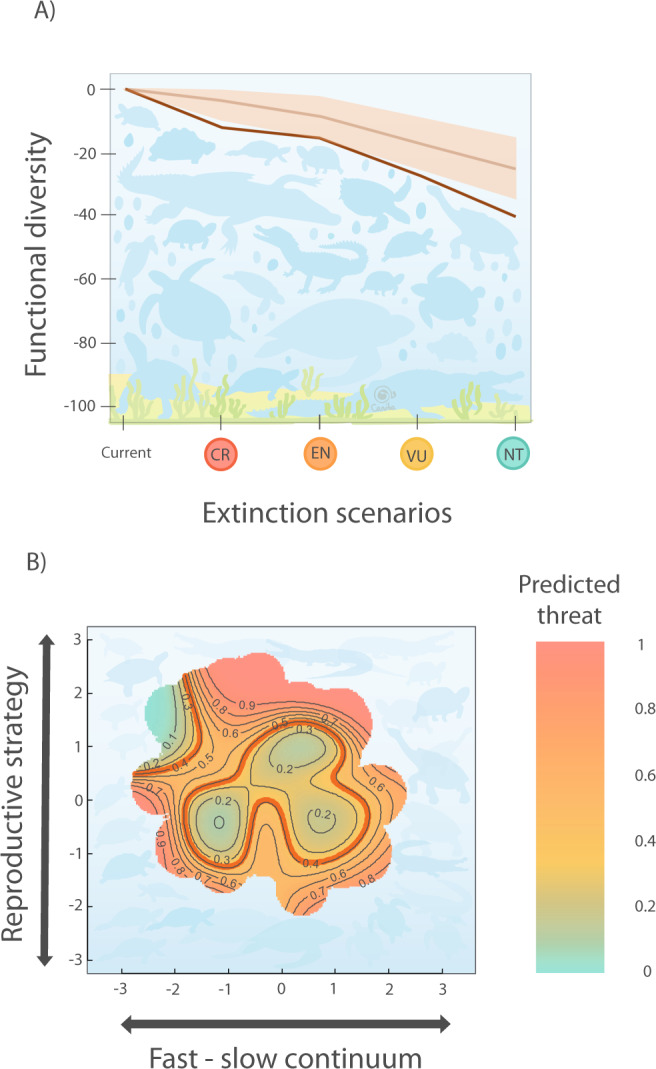
The life history strategies of Testudines and Crocodilia predict their vulnerability to extinction due to human threats. **A** Loss of functional diversity under extinction scenarios of threatened species. CR critically endangered, EN endangered, VU vulnerable, NT near threatened. The loss of functional diversity is expressed as a percentage of the current functional diversity in the life history strategies of species of the order Testudines and Crocodilia (Fig. 1). We simulate the loss of functional diversity by removing all species from the IUCN category in a progressive manner, by degree of “endangerment”, removing first the species with a higher risk of extinction (i.e. -CR), then continuing progressively to remove species from the categories with lower threatened risks (-EN scenario: removing CR and EN species; -VU scenario: removing CR, EN and VU; -NT scenario: removing CR, EN, VU and NT). This loss of diversity is represented by a dark red line. For each scenario, we compare the loss of functional diversity with 999 iterations of a null model where threatened species are randomly selected among the 259 species in the analyses. The 999 randomisations are represented by a orange shadow defined by the 5th and 95th percentiles of functional diversity loss, with the orange line representing the 50th percentile; **B** Risk of extinction in the functional space of Testudines and Crocodilia species. Probability of species being classified as high-risk of extinction (i.e., CR and EN IUCN status) according to GAMs (with binomial distribution) using the position of each species in the two-dimensional functional space as predictors. Blue tones indicate lower risk of extinction, whereas red tones indicate higher risk of extinction. *p* value of the GAM model is 0.04 and *χ*^2^ is 28.59. The red contour lines indicate the average threat probability (proportion of species classified as threatened in the group). Here, we consider only the 214 out of the 259 available species of testudines and crocodilians for which the threat status is known.

The extinction of threatened species of testudines and crocodilians would result in the loss of a quarter of the global functional diversity of these taxa. To estimate the effect of the potential extinctions of threatened species on the existing amount of functional diversity in these groups, we use the IUCN Red List categories of our species and simulate an accumulative loss of threatened species (i.e., in the first scenario “-CR”, we remove the *Critically Endangered* species only; in the “-EN” scenario, we remove Endangered and Critically Endangered species, and so on; see “Methods”). If all threatened species disappeared (*Critically Endangered*, *Endangered*, and *Vulnerable* species*;* “-VU” scenario, Fig. 2A), the functional diversity would decrease by 26.8%.

The loss of functional diversity due to the extinction of threatened species is greater than expected by chance. We compare the potential loss of functional diversity, considering the number of threatened species in each extinction scenario, with a simulated loss of functional diversity where the identity of the threatened species is randomised within the pool of species. The resulting loss of functional diversity due to the extinction of threatened species is greater than 95% of randomisations for most scenarios (Fig. 2A). In the “-CR” scenario, the loss of functional diversity (−12.67%) is four times greater than in randomisations (−3.82% [−0.4%, −9.69%] 5% and 95% CI). This finding suggests that most of the Critically Endangered species of testudines and crocodilians show unique life history strategies, such as the pancake tortoise (*Malacochersus tornieri* max. lifespan 25.9 years, one egg/clutch, one clutch/year).

Unique or less redundant species strategies of testudines and crocodilians are more vulnerable to vanish following the extinction of threatened species. To identify differences in extinction risk between the life history strategies, we map extinction risk in the life history trait spectra via generalized additive models (GAMs), using the IUCN Red List category of each species^5^. The position of each species along the trait spectrum is significantly correlated with its extinction risk when considering high-risk species as Critically Endangered and Endangered (*p* = 0.039; Fig. 2B). Using this grouping, high-risk species show distinct positions in the life history functional space, with species with lower functional redundancy located around the periphery and likely being more threatened. Only species located at the centre of the functional space and those with short maximum lifespan and smaller clutch sizes but with greater number of clutches per year show a lower probability of being threatened (Fig. 2B).

### Habitat degradation as the main threat

**Fig. 3:**
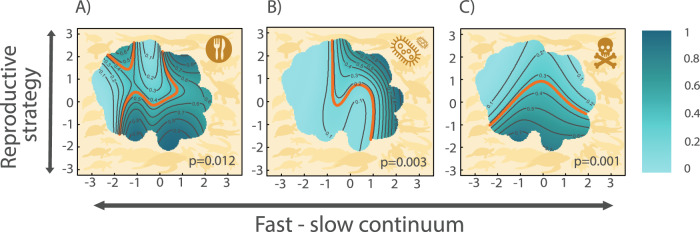
The life history strategies of Testudines and Crocodilia predict the vulnerability to extinction due to local consumption, interaction with invasive species and diseases, and pollution. Other risks, such as climate change, global trade, or habitat degradation, do not show a significant relation with the examined species’ life history strategies (Supplementary Fig. 1). Probability of species being affected by: **A** local consumption, **B** interaction with invasive species and diseases, and **C** pollution, according to GAMs (with binomial distribution) using the position of species in the two-dimensional functional space (Fig. 1) as predictors. Light tones indicate lower risk of extinction due to each threat, whereas dark tones indicate higher risk. *p* values associated with each GAM are shown at the bottom-right corner of each panel, and *χ*^2^ is 27.36 for model **A**; 25.92 for **B** and 19.22 for **C**. The red contour lines indicate the average threat probability. Here, we consider only species whose threats are known (*n* = 251 species).

The life history strategies of Testudines and Crocodilia can predict vulnerability to extinction due to three of the six analysed threats: local consumption, interaction with invasive species and disease, and pollution (Fig. 3). To explore the differential effects of global and local threats on the functional spectra of testudines and crocodilians, we analyse the probability of being affected by each threat separately using GAMs (more results in Supplementary Fig. 1). Local consumption (Fig. 3A) is associated with increased extinction risk in species with higher clutch size such as sea turtles (e.g., *Caretta caretta*), and higher maximum lifespans, such as the radiated tortoise (*Astrochelys radiata*) or the Orinoco crocodile (*Crocodylus intermedius*). In contrast, interaction with invasive species and diseases are associated with threatened species with slow life histories (Fig. 3B), such as the desert tortoise (*Gopherus agassizii*). Finally, pollution primarily is associated with species with higher reproductive output (i.e., higher clutch sizes), such as freshwater turtles like *Chelydra serpentina* or crocodilians like the saltwater crocodile, *Crocodylus porosus*, (Fig. 3C).

**Fig. 4:**
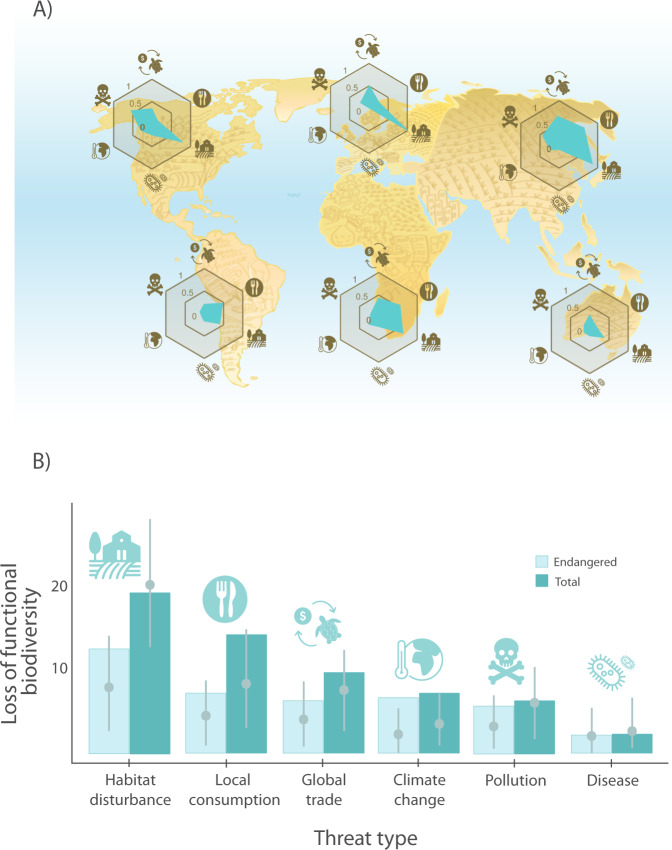
Habitat degradation is the main threat for the functional diversity, especially in the north hemisphere. **A** Ratio of species affected by each threat type per continent. The ratio is only estimated for species whose threats are known at the continent level: North America, *n* = 65 (34% are threatened); Europe, *n* = 4 (0%); Asia, *n* = 66 (83%); South America, *n* = 57 (44%); Africa, *n* = 39 (41%); Oceania, *n* = 14 (36%). **B** Simulated loss of functional diversity of Testudines and Crocodilia under extinction scenarios by threat (*N* = 251). The loss of functional diversity is expressed as a percentage of the total spectra of functional diversity of species, as detailed in Fig. 1. We simulate the loss of functional diversity by removing all species affected by specific threats, in light blue colour removing only threatened species (i.e. Critically Endangered [CR], Endangered [EN] and Vulnerable [VU] as per the IUCN Red List) and in dark blue removing all the species affected (threatened or not). For each scenario, we compare the loss of functional diversity with 999 iterations of a null model where the same number of species were randomly selected among all 251 species. The 999 randomisations are represented for each threat as a grey dot for the 50th percentile, with grey whiskers representing the 5th and 95th percentiles.

Among examined threats, the extinction of species affected by habitat degradation results in the greatest loss of functional diversity, especially in the northern hemisphere (Fig. 4A). To estimate the effect of specific threats on the loss of functional diversity, we simulate the extinction of species affected by each threat to calculate the percentage of loss of functional diversity (Fig. 4B). We compare the potential loss of functional diversity by each threat, with a simulated scenario where the identity of the species affected by each threat is randomised within the pool of species. Most of the effects of loss of functional diversity due to the six threats considered are greater than expected by random extinctions, especially for climate change, whose effect is beyond the 95% percentile for random simulations (Fig. 4B). Only habitat degradation and interaction with invasive species and diseases show similar or lower values of loss of functional diversity than expected by random simulations (Fig. 4B).

## Discussion

Here, we demonstrate that threatened species of turtles and crocodilians show great deal of functional disparity. Toussaint et al.^6^, exploring 5686 species of reptiles, found a loss of functional diversity linked to the extinction of the threatened species corresponding to less than 10% of the total functional spectra. Here, focussing on Testudines and Crocodilia, for which much information has been missing to date^33^, we find twice the loss of functional diversity. By linking this information to their conservation status, we demonstrate that global and local threats are exposing the functional diversity of these already highly threatened taxonomic groups to great peril. Using the largest collection of trait data to date for turtles and crocodilians, we find that the threatened species show unique life history strategies. Losing these species due to extinction will result in a greater reduction of functional diversity and potentially severe ecological consequences^34^ due to the ecosystem services they provide, such as the high values of biomass contribution of turtles (e.g., red-eared slider turtle, *Trachemys scripta*^35^), mineral cycling and bioaccumulation (e.g., *Chelydra serpentine*^36^), or seed dispersal (e.g., Galapagos tortoises, *Chelonoidis niger* complex^37^). Moreover, our results could be conservative because the 131 threatened species in our study show a borderline significant trend towards having more missing traits than non-threatened species (Kruskal–Wallis test: *p* = 0.04, *χ*^2^ = 19.96, df = 2; Supplementary Methods. Missing Data), and imputation methods are known to impute more similar traits with the existing data (Supplementary Methods. Imputation validation).

Local disturbances such as habitat degradation and unsustainable local consumption are the main threats for the functional diversity of the studied species. Habitat degradation affect most species of turtles and crocodilians, especially in North America and Europe (Fig. 4A), independent of their life history strategies. Therefore, habitat protection for these species is key to preserve the functional diversity^38^. In fact, Mittermeier et al.^39^. highlighted that the protection of only 16 hotspots of turtle biodiversity would successfully encompass 83% of these species. On the other hand, we find that the second local threat for the functional diversity of Testudines and Crocodilia is unsustainable local consumption. Our results predict the extinction of species with higher clutch sizes (e.g., such as sea turtles, whose eggs are consumed in high quantities^40^) and long-lived species (linked with larger size, which typically provides more meat^41^). Unsustainable local consumption of turtles is one of the most important threats in Asia, South America, and Africa (Fig. 4A). For example, in China, one million turtle farms are the primary purchasers of turtles caught in the wild, including several threatened species^42^.

Global threats such as unsustainable or illegal international trade and climate change can also have important effects in reducing the functional diversity of Testudines and Crocodilia. The global trade of these species is the third most important threat for their functional diversity, affecting tortoises and turtles mainly for their use as pets^43^ and crocodilians for their skin^44,45^. On the other hand, although climate change affects fewer species than other threats, turtles and crocodilians with unique life history strategies could disappear according to our projections^46^. Indeed, the consequences of climate change for these species are found worldwide, including extreme droughts by compromising the fitness of the populations^47,48^, flood nesting sites by rising sea levels^49^, coral bleaching due to increasing in temperatures, affecting feeding areas of sea turtles^50^ and the imbalance of sex-ratios in populations of species with temperature-dependent sex determination^51^, such as the threatened leatherback turtle (*Dermochelys coriacea*)^52^.

Our study does not capture all human activities that can impact species’ life history strategies. Importantly, biases exist regarding the assessment of threats^53^; for instance, some threats, like climate change, lack information for many species because it is a relatively recent problem^53^. As such, we deem our findings to be conservative, and we argue that, when more threat information becomes available, we will likely—and unfortunately—see stronger effects in the loss of functional diversity of our examined species. For instance, although we have summarised the breadth of testudines and crocodilians’ threats to extinction, threat interactions, which remain largely unknown in these taxonomic groups, have been reported to have synergistic effects on the loss of species, as was described for mammals^54^ and birds^55^. Moreover, examining the ecological functions of each species in higher resolution, by examining their diets or habitat breadth (i.e., generalists vs. specialists), could improve the available information of ecological function^56^.

Protecting threatened species with unique life history strategies is key to conserving their functional diversity^34^ and ecosystem services they provide^35^. Our findings support previous studies that suggest that conservation policies should consider together the taxonomic and functional dimensions of biodiversity^5^ to implement targeted and more effective conservation actions in the context of a global biodiversity crisis^6^. The IUCN Red List of Threatened Species may include information about population trends, distribution area, threats, and abundance. The inclusion of functional diversity could greatly aid international, national, and local efforts to preserve functionally unique species, and recently, new indexes have been developed to include some aspects of functional diversity. The IUCN Green Status of Species^57,58^, a new part of the Red List assessment, quantifies species recovery and explicitly includes ecological function^59^. Although we have used a set of traits based on demography, other traits based on species ecology could offer complementary results for the conservation species and the ecosystem services of this taxonomic group. Incorporating the functional diversity of life history strategies in conservation assessments constitutes a promising approach to help prioritise conservation efforts to maintain high levels of functional diversity against current and future threats.

## Methods

### Life history traits

To describe the life cycle of species in the orders Testudines and Crocodilia, we used life history trait data. Life history traits quantify how the life cycle of a species and its underlying vital rates: survival, development, and reproduction have been shaped by natural selection to optimise its performance^60^. We obtained such traits from published literature, open access databases, and via direct researcher contributions^61^. In some cases, life history trait information was derived from the demographic information of different databases and datasets. These data were obtained from the COMADRE Animal Matrix Database^62^, DATLife Database^63^, Amniote Life History Database^64^, and published reviews^65–67^ (more information about data selection in Supplementary Methods and Supplementary Data 1).

To quantify species’ life history strategies, we selected six life history traits that encompass detailed information regarding the timing, intensity, frequency, and duration of demographic processes across the life cycle of any species^7^. The life history traits chosen were adult survival (*Sa*), juvenile survival (*Sj*), maximum lifespan (*ML*), age at sexual maturity (*Lα*), mean number of clutches per year (*CN*), and clutch size (*CS*). These traits are well-established metrics in comparative demographic analyses, and their emerging syndromes typically explain high variance (~75%) in life history strategies of multicellular organisms^5,7,8,16,29,31^. The data obtained from the aforementioned sources encompass 259 species (we considered the species in ref. ^21^) with at least one of these six traits (more information about data collection/estimation in Supplementary Methods). To avoid collinearities in the models, we also evaluated the correlation between each life history trait, but we did not find strong correlations (Supplementary Tables 3 and 4).

### Phylogeny and data imputation

To identify and quantify the potential role of evolution in shaping our species’ life history strategies, we performed phylogenetic comparative analyses. We used a species‐level phylogenetic tree recently published for Testudines and Crocodilia^21^ to both impute the missing data and account for the effect of evolutionary constraints in the observed life history strategies (we used the number of species described in ref. ^21^). Briefly, to build the phylogenetic tree, 14 mitochondrial loci and six nuclear loci were sampled for 357 species of extant or recently extinct turtle and tortoises, and the 27 species described crocodilian species^68^. Colston et al.^21^ computed Maximum Likelihood trees with RaxML^69^ and used phylogenetic multiple imputation to fill the gaps of 17 out of 384 (<5%) species in the tree. Further details on the methods for tree construction are available in ref. ^21^. We selected the demographic information of species when information about the phylogeny is available, to perform phylogenetic comparative analyses^70,71^, the final dataset included 259 species of Crocodilia (23) and Testudines (236) with this resource.

Demographic data of reptiles are scarce and contain missing values^33^ (Supplementary Fig. 2). As principal component analyses require full data (i.e. non-gappy data)^72,73^, we carried out phylogenetic imputation to fill in the gaps in the life history traits for the studied species (see Supplementary Methods. Missing data). To impute the missing life history traits in our dataset, we used the R package *mice*^74^, which uses multiple imputation and the addon-*phylomice* to include phylogenetic information. Multiple imputation has been shown recently to correctly impute missing data in similar datasets better than other available approaches^75^. Briefly, this approach uses Fully Conditional Specification (FCS) of the imputation model, which specifies the multivariate imputation model on a variable-by-variable basis by a set of conditional densities, one for each incomplete variable^74^. As suggested by previous studies^76^, we included three more traits in the imputation analyses in order to have more robust estimations of the imputed data (we included body weight and body size, which is available for all the species, and incubation time available for 16% of the species from Amniote Database). To quantify uncertainty in the imputation method, we then created 40 imputed datasets and ran analyses on each separately. We run 40 datasets because the percentage of gaps in the data were 38% for all the traits added in the imputation. Here, we used the predictive mean matching (*pmm*) method, which imputes missing values by means of the nearest-neighbour with distance based on the expected values of the missing variables conditional on the observed covariates. Moreover, we chose *m* = 1 for the number of multiple imputation (*m*) and *maxit* = 15 for the number of iterations to reach an adequate convergence in the Markov chains and the computation time. Our approach used the *pmm* with phylogenetic information. Thus, we evaluated the phylogenetic signal of each life history trait. We first estimated the phylogenetic signal for each trait separately using Pagel’s *λ*, which describes the strength of phylogenetic relationships on trait evolution under a Brownian motion model^77^, we estimated phylogenetic signal for raw data and imputed data (see Supplementary Methods. Phylogenetic Signal). Pagel’s *λ* ranges between 0, when the patterns in the traits cannot be explained by the employed phylogeny, and 1 when the observed patterns in traits are tightly correlated with the placement of species in the phylogeny. Most of the selected traits showed a strong phylogenetic signal (>0.8; see Supplementary Methods. Phylogenetic signal), so we used the imputed values for traits in further analyses. We evaluated the similarity between imputed and non-imputed data using density plots for each trait. Moreover, we analysed the position in the functional spectra using Procrustres analyses between imputed data and a subset where a maximum of two traits were imputed (see Supplementary Methods. Imputation validation).

### Exploring the relation between the axes of life history strategies

To identify potential differences between the patterns of association among life history traits for Testudines and Crocodilia species while simultaneously assessing non-independence of lineages, we used a phylogenetically informed PCA (*p*PCA)^78^. *p*PCA is a multivariate analysis that reduces the number of variables of interest due to their plausible correlation. The *p*PCA considers the correlation matrix of species’ traits while accounting for phylogenetic relationships and estimate Pagel’s *λ*. The *p*PCA was estimated using the R package *phytools*^71^, assuming a Brownian motion model of evolution^70^. Life history trait data were log‐transformed to fulfil normality assumptions of PCA and z‐transformed to mean = 0, and SD = 1^79^. We used the Kaiser criterion^80^ after optimisation through varimax rotation to determine the number of axes necessary to explain a substantial amount of variation. Namely, the axes we retained had an associated eigenvalue >1. We run 40 *p*PCA according to the 40 imputed datasets, we pooled the results for scores and loadings (uncertainty in the estimates are in Supplementary Tables 1 and 2 and Supplementary Data 2).

As body mass is strongly correlated with life history traits^11,16,29^, we accounted for this potential effect in our multivariate analyses too. Out of the multiple possible ways to account for body mass in life history analyses, we used the residuals of phylogenetic regression between body size and each trait^78^. We collected most of adult body mass (g) data from the studies by Myhrvold et al.^64^ and Colston et al.^21^, moreover, we collected the remained data from previous literature for all our 259 species (see Supplementary Methods. Body mass data and Supplementary Data 3).

### Estimating the functional spectra

To describe the probabilistic distribution of the species within the functional spaces, we used the main axes of life history strategy variation by *p*PCA corrected by size (i.e., residuals of regressions). We performed multivariate kernel density using the “*TPD*” (Trait probability density) and “*ks*” R packages^81–84^ to estimate the functional spectra. The kernel was a multivariate normal distribution for each species centred in the location of the species in *p*PCA and bandwidth chosen using unconstrained bandwidth selectors from the “*Hpi*” function in the “*ks*” package^85^. The grouped kernels for all species drive into the continuous TPD function^32,84^. According to Carmona et al.^5^ and Toussaint et al.^6^, we divided the continuous functional space into a two-dimensional grid composed of 200 equal sized cells per dimension. We estimated the value of the TPD function for the 40,000 cells. The value of the TPD function represent the density of species in that particular region of the functional space (i.e., species with similar life history strategy). Results of TPD were represented graphically with the contours containing 50, 60, 70, 80, 90, and 99% of the total density of species.

### Threats to the studied species

To identify causes of recent declines in Testudines and Crocodilia species we reviewed published information. The main threats described for these species were (1) habitat loss, fragmentation, and degradation^86^, (2) over-collection of individuals and their eggs for food consumption^87^, (3) unsustainable or illegal international trade, as well as over-collection for the trade in medicines^88^, (4) climate change^89^; (5) interaction with invasive species and diseases^90,91^. We collected the information on the threats to each species from Stanford et al.^26^, Bonin et al.^92^ and the IUCN Red List^23^. We did dichotomous variables when one of these threats were mentioned. We were not able to quantify the effect of the threat, because effect size is only available for the Critically Endangered species^26^. To convert the threat descriptions of the Red List into our broader categories, we used the following process: for habitat degradation we considered the Red List threat classifications “residential & commercial development”, “agriculture & aquaculture” and “natural system modifications”; for climate change the classification “climate change and extreme weather”; and for disease the classification “invasive and other problematic species, genes & diseases”. We were able to categorise the threats in 251 species (Supplementary Data 4).

### Effects of extinctions of threatened species on global functional diversity

To identify the loss of functional diversity by threatened species extinctions, we simulated the disappearance of species according to their Red List category (IUCN, 2020). We analysed 214 out of the 259 available species of chelonians and crocodilians for which the threat status is known. The species classified among the threatened species categories (i.e. Critically Endangered (CR, *N* = 46), Endangered (EN, *N* = 36) and Vulnerable (VU, *N* = 49) have a higher risk of extinction than the rest of species: Near Threatened (NT, *N* = 25), Least Concern (LC, *N* = 51); the extinction risk of Data Deficient (DD = 7) species is unknown. In our analysis, we removed threatened species and estimated the resulting shifts in functional diversity according previous approaches^6^. We used this method to evaluate the effect of the loss of species according to the conservation status, independently of the extinction probability. Firstly, we removed the species with a higher risk of extinction (-CR), then we removed successively the species with lower risks (-EN includes the extinction of species classified as a CR and EN, -VU includes CR, EN and VU, and -NT includes CR, EN, VU and NT). These extinction scenarios represented a gradient of extinction risk from the persistence of all species to a more dramatic scenario where all threatened species (including the NT species) went extinct. We compared the TPD function considering all the species assessed by IUCN (current spectra of functional diversity), and the TPD function after removing the species in the different scenarios. We can compare TPD functions because they are probability density functions, which means that they integrate to 1 across the whole functional space, regardless of the number of species considered^32^. To reduce the potential effect of outliers in the functional space, we applied a quantile threshold of 99%. We quantify how much of the functional spectra is lost after the extinction scenarios by estimating which functional space cells become empty after extinctions. In other words, the difference between the occupied functions space for all the species and the functional space after the simulated extinctions. The difference in the amount of space occupied before and after extinctions (TPD function of the current spectra − TPD function after removing the threatened species) represented the loss of functional diversity for Testudines and Crocodilia. We calculated this loss in the functional space for all the extinction scenarios (-CR, -EN, -VU, and -NT).

To assess if the impacts of each extinction scenario were different from what would be expected if extinction risk is not related to life history traits, we also compared the observed changes in functional diversity to a null model where the extinct species were randomly selected within the pool of species. For each scenario of extinction risk, we compared the functional diversity to 999 losses of functional diversity where the same number of threatened species were randomly selected among the pool of species. This strategy allowed us to understand whether the extinction in the different scenarios reduces the functional diversity more or less than expected. For each scenario, we created 999 TPD functions simulating cases in which the same number of species were lost at random from the total set of IUCN-assessed species. We compared the 5% and 95% percentile of the random simulation with the value of loss of functional diversity per each extinction scenario to estimate if the values were significantly different. In this case, higher than expected reductions in functional diversity would mean that the species that are going extinct in the considered scenario are unique in terms of their life history strategies, whereas lower than expected reductions would imply that the species going extinct are mostly functionally redundant.

After examining the loss of functional diversity, we plotted the extinction risk of species within the functional spaces, using the species assessed by IUCN. Using binomial smoother-based GAM^93^ with the R package *mgcv*^94^, we analysed the relationship between extinction risk (1: high-risk and 0: non-threatened or low-risk) and the position in the functional space (PC axes). We then represented the predictions of the models (including the 95% confidence intervals of the means) to visually examine how life history strategies affect the probability of species being threatened^5^.

### Effects of anthropic threats on global functional diversity

To identify the effect of the different threats in the functional spectra, we simulated scenarios where extinctions were based upon species reported as affected by specific threats. We did two comparisons: (1) the TPD function considering all the species (current spectra of functional diversity) and TPD function after removing the species affected by specific threat (habitat degradation, trade, local consumption, climate change or interaction with invasive species and diseases), and (2) the TPD function considering all the IUCN-assessed species, and the TPD function after removing the threatened species (CR, EN, or VU) affected by each specific threat. Using a comparison between TPD functions similar to the one explained above, we estimated the potential loss of functional diversity attributable to each threat and evaluated the differences between them. We also compared the observed changes in functional diversity using a null model to assess if the impacts of each extinction scenario were different from what would be expected by chance. For each threat, we compared the loss of functional diversity with the 999 losses of functional diversity where the same number of species were randomly selected among the pool the species. We did these comparisons both considering the extinction of all the species affected by each threat and considering only the extinction of threatened species.

To visualise the relationship between life history strategies and threats, we mapped the probability of a species being affected by a specific threat within the functional spaces. We used binomial smoother-based GAM^93^ with the R package *mgcv*^94^ to identify the relationship between being affected by each threat (1: affected and 0: nonaffected) and the position in the functional space (PCA axes). We represented the predictions of the models (including the 95% confidence intervals of the means) when the relation between threat and life history strategy was significant.

### Reporting summary

Further information on research design is available in the Nature Portfolio Reporting Summary linked to this article.

## Supplementary information

Supplementary Information

Description of Additional Supplementary Files

Supplementary data 1

Supplementary data 2

Supplementary data 3

Supplementary data 4

Supplementary Software

Reporting Summary

## Data Availability

All data generated in this study are provided in the Supplementary Information and Supplementary Software.

## Code availability

All code generated in this study are provided in the Supplementary Software.
