# Supplementary material for: Anthropogenic impacts on threatened species erode functional diversity in chelonians and crocodilians
Source: Nat Commun. 2023 Mar 28;14:1542. doi: 10.1038/s41467-023-37089-5 (PMC10050202; doi:10.1038/s41467-023-37089-5)
Supplement: Supplementary file 1 — Supplementary Information [file 41467_2023_37089_MOESM1_ESM.pdf]

Supplementary Information

Anthropogenic impacts on threatened species erode functional diversity in chelonians and crocodilians

Rodríguez-Caro, R.C., Graciá, E., Blomberg, S. P., Cayuela, H., Grace, M., Carmona, C.P., Pérez-Mendoza, H.A., Giménez, A., Salguero-Gómez, R.

Index

Supplementary Tables **P. 2**

Table S1 P. 2

Table S2 P. 3

Supplementary Figure **P. 9**

Figure S1 P. 9

Supplementary Methods **P. 10**

Data selection P. 10

Missing data P. 14

Imputation validation P. 17

Body mass data P. 22

Phylogenetic signal P. 31

Supplementary References **P. 33**

Supplementary Tables

Supplementary Table 1. Loadings of phylogenetically-corrected principal component analysis (*p*PCA), also corrected by body mass, for the 236 species of Testudines and 23 species of Crocodilia examined in this study. Only the first two PC axes are shown, as only they raised associated eigenvalues >1, indicating that they explain sufficient observed variation in life history traits[1]. Bold numbers indicate loading absolute values >0.50. Outputs correspond to the mean values of 40 imputed data sets. Pagel's λ indicates the extent to which patterns are explained (=1) or not (=0) by phylogenetic relationships. Variance estimates (±) correspond to standard deviation.

Life history trait	Symbol	PC1	PC2
Adult survival	*Sa*	0.03 ± 0.08	0.01 ± 0.12
Juvenile survival	*Sj*	0.04 ± 0.17	-0.06 ± 0.33
Age at sexual maturity	*Lα*	0.03 ± 0.09	-0.03 ± 0.10
Clutch size	*CS*	**0.56 ± 0.07**	**-0.767** ± 0.08
Mean number of clutches per year	*CN*	-0.13 ± 0.08	0.191 ± 0.14
Maximum lifespan	*ML*	**0.92 ± 0.04**	0.377 ± 0.10
**Proportion of variance explained**		39.1% ± 1.9%	23.1% ± 1.2%
**Cumulative proportion of variance explained**		39.1%	62.2%
**Pagel's λ**		0.67 ± 0.03	

Supplementary Table 2. Scores of phylogenetically-corrected principal component analysis (*p*PCA), also corrected by body mass, for the 236 species of Testudines and 23 species of Crocodilia examined in this study. Only the first three PC axes are shown. Outputs correspond to the mean values of 40 imputed data sets. Variance estimates (±) correspond to standard deviation.

Species	PC1	PC2
Acanthochelys pallidipectoris	-0.92 ± 0.53	0.75 ± 0.28
Acanthochelys spixii	-1.59 ± 0.10	0.03 ± 0.25
Aldabrachelys gigantea	1.32 ± 0.15	1.09 ± 0.19
Alligator mississippiensis	1.19 ± 0.06	-0.18 ± 0.15
Alligator sinensis	0.80 ± 0.05	0.10 ± 0.16
Amyda cartilaginea	-1.24 ± 0.11	-0.81 ± 0.25
Apalone ferox	0.10 ± 0.11	0.08 ± 0.18
Apalone mutica	-0.97 ± 0.07	-0.46 ± 0.18
Apalone spinifera	0.47 ± 0.07	0.03 ± 0.10
Astrochelys radiata	0.41 ± 0.17	1.54 ± 0.17
Astrochelys yniphora	0.33 ± 0.21	1.70 ± 0.24
Batagur baska	-0.23 ± 0.11	-0.41 ± 0.15
Batagur borneoensis	0.60 ± 0.08	0.75 ± 0.21
Batagur dhongoka	-1.09 ± 0.12	-1.22 ± 0.27
Batagur kachuga	-0.06 ± 0.50	-0.06 ± 0.34
Batagur trivittata	-0.12 ± 0.46	-0.44 ± 0.28
Caiman crocodilus	0.73 ± 0.05	-0.07 ± 0.09
Caiman latirostris	-0.06 ± 0.11	-0.70 ± 0.13
Caiman yacare	0.74 ± 0.07	-0.12 ± 0.14
Caretta caretta	1.52 ± 0.11	-0.99 ± 0.18
Carettochelys insculpta	0.35 ± 0.05	-0.29 ± 0.11
Chelodina longicollis	0.12 ± 0.08	0.05 ± 0.18
Chelodina mccordi	-0.68 ± 0.46	0.45 ± 0.29
Chelodina novaeguineae	0.08 ± 0.12	0.11 ± 0.21
Chelodina reimanni	0.06 ± 0.10	0.16 ± 0.22
Chelonia mydas	1.50 ± 0.12	-0.97 ± 0.19
Chelonoidis becki	1.13 ± 0.18	1.42 ± 0.25
Chelonoidis carbonarius	-0.18 ± 0.15	1.13 ± 0.17
Chelonoidis chathamensis	1.13 ± 0.19	1.40 ± 0.23
Chelonoidis chilensis	-1.16 ± 0.13	1.04 ± 0.21
Chelonoidis darwini	1.11 ± 0.17	1.43 ± 0.23
Chelonoidis denticulatus	0.26 ± 0.14	1.08 ± 0.18
Chelonoidis duncanensis	1.84 ± 0.11	0.12 ± 0.24
Chelonoidis hoodensis	1.14 ± 0.17	1.40 ± 0.23
Chelonoidis porteri	1.08 ± 0.19	1.49 ± 0.25
Chelonoidis vicina	1.08 ± 0.18	1.50 ± 0.24
Chelydra rossignonii	0.63 ± 0.06	-0.38 ± 0.13
Chelydra serpentina	0.64 ± 0.06	-0.39 ± 0.10
Chersina angulata	-2.06 ± 0.19	1.62 ± 0.25
Chitra indica	1.30 ± 0.48	-1.01 ± 0.30

Chrysemys dorsalis	0.23 ± 0.16	0.81 ± 0.28
Chrysemys picta	0.26 ± 0.15	0.79 ± 0.19
Claudius angustatus	-1.03 ± 0.10	0.39 ± 0.23
Clemmys guttata	-0.42 ± 0.14	0.56 ± 0.23
Crocodylus acutus	0.34 ± 0.09	-0.58 ± 0.15
Crocodylus intermedius	1.10 ± 0.10	-0.16 ± 0.22
Crocodylus johnsoni	-0.53 ± 0.08	-0.04 ± 0.16
Crocodylus mindorensis	-0.14 ± 0.08	-0.32 ± 0.16
Crocodylus moreletii	1.03 ± 0.08	-0.03 ± 0.16
Crocodylus niloticus	0.99 ± 0.07	-0.37 ± 0.16
Crocodylus novaeguineae	-0.02 ± 0.10	-0.52 ± 0.14
Crocodylus palustris	0.12 ± 0.08	-0.26 ± 0.13
Crocodylus porosus	0.62 ± 0.09	-0.60 ± 0.16
Crocodylus rhombifer	0.34 ± 0.09	-0.29 ± 0.15
Crocodylus siamensis	-0.15 ± 0.09	-0.52 ± 0.14
Cuora amboinensis	-1.00 ± 0.23	2.12 ± 0.23
Cuora aurocapitata	-0.92 ± 0.56	0.89 ± 0.25
Cuora bourreti	-1.24 ± 0.16	1.10 ± 0.21
Cuora flavomarginata	-1.23 ± 0.13	1.11 ± 0.23
Cuora galbinifrons	-1.08 ± 0.40	1.36 ± 0.25
Cuora mccordi	-1.25 ± 0.40	1.46 ± 0.32
Cuora mouhotii	-1.03 ± 0.50	1.16 ± 0.35
Cuora picturata	-1.19 ± 0.49	1.40 ± 0.29
Cuora trifasciata	-0.88 ± 0.16	1.49 ± 0.21
Cuora yunnanensis	-0.47 ± 0.44	0.17 ± 0.27
Cyclanorbis senegalensis	-1.50 ± 0.48	-0.64 ± 0.85
Cyclemys dentata	-1.33 ± 0.12	0.74 ± 0.23
Cycloderma frenatum	0.15 ± 0.62	-0.06 ± 0.34
Deirochelys reticularia	-0.45 ± 0.09	0.18 ± 0.19
Dermatemys mawii	-1.25 ± 0.09	0.08 ± 0.17
Dermochelys coriacea	1.22 ± 0.14	-0.36 ± 0.25
Elseya dentata	-0.84 ± 0.08	0.68 ± 0.18
Emydura macquarii	-0.42 ± 0.07	-0.14 ± 0.13
Emydura subglobosa	-0.42 ± 0.40	0.23 ± 0.25
Emydura tanybaraga	-0.59 ± 0.62	0.15 ± 0.35
Emys orbicularis	1.00 ± 0.13	0.88 ± 0.19
Eretmochelys imbricata	0.38 ± 0.15	-1.63 ± 0.17
Erymnochelys madagascariensis	-0.13 ± 0.40	-0.42 ± 0.24
Gavialis gangeticus	1.25 ± 0.10	0.04 ± 0.20
Geochelone elegans	-0.71 ± 0.12	0.68 ± 0.15
Geochelone platynota	-0.71 ± 0.10	0.67 ± 0.17
Geoclemys hamiltonii	-0.47 ± 0.10	-0.22 ± 0.21
Geoemyda japonica	-1.34 ± 0.53	1.75 ± 0.26
Geoemyda spengleri	-1.31 ± 0.45	1.74 ± 0.30
Glyptemys insculpta	0.36 ± 0.10	0.58 ± 0.16
Glyptemys muhlenbergii	-0.32 ± 0.15	0.95 ± 0.28
Gopherus agassizii	0.19 ± 0.15	0.99 ± 0.19
Gopherus berlandieri	-0.14 ± 0.18	1.47 ± 0.20

Gopherus flavomarginatus	-2.31 ± 0.11	0.25 ± 0.43
Gopherus polyphemus	0.24 ± 0.11	0.81 ± 0.11
Graptemys barbouri	-0.26 ± 0.13	0.45 ± 0.26
Graptemys caglei	-1.32 ± 0.11	0.68 ± 0.21
Graptemys ernsti	-0.61 ± 0.46	0.49 ± 0.27
Graptemys flavimaculata	-0.24 ± 0.11	0.70 ± 0.11
Graptemys geographica	-0.45 ± 0.10	-0.21 ± 0.19
Graptemys gibbonsi	-0.81 ± 0.10	0.44 ± 0.20
Graptemys nigrinoda	-0.21 ± 0.12	0.62 ± 0.18
Graptemys oculifera	-0.12 ± 0.09	0.48 ± 0.18
Graptemys ouachitensis	-0.07 ± 0.10	0.33 ± 0.17
Graptemys pearlensis	-0.72 ± 0.58	0.37 ± 0.3
Graptemys pseudogeographica	-0.09 ± 0.11	0.33 ± 0.17
Graptemys pulchra	-0.79 ± 0.11	0.46 ± 0.14
Graptemys versa	-0.21 ± 0.12	0.52 ± 0.22
Hardella thurjii	-0.77 ± 0.53	0.20 ± 0.88
Heosemys annandalii	-1.32 ± 0.48	-0.21 ± 0.77
Heosemys grandis	-0.57 ± 0.12	0.85 ± 0.21
Heosemys spinosa	-1.45 ± 0.20	1.85 ± 0.27
Homopus areolatus	-0.68 ± 0.13	0.94 ± 0.21
Homopus femoralis	-1.04 ± 0.40	1.18 ± 0.27
Hydromedusa maximiliani	-0.95 ± 0.51	-0.35 ± 0.91
Hydromedusa tectifera	-1.67 ± 0.08	0.32 ± 0.24
Indotestudo elongata	-1.19 ± 0.13	0.46 ± 0.20
Indotestudo forstenii	-0.54 ± 0.15	0.96 ± 0.17
Indotestudo travancorica	-1.21 ± 0.14	0.44 ± 0.23
Kinixys belliana	-1.03 ± 0.18	1.42 ± 0.25
Kinixys erosa	-0.97 ± 0.15	1.16 ± 0.25
Kinixys homeana	-1.00 ± 0.53	0.96 ± 0.29
Kinixys lobatsiana	-0.93 ± 0.11	1.24 ± 0.22
Kinixys natalensis	-0.93 ± 0.12	1.22 ± 0.21
Kinixys nogueyi	-0.97 ± 0.18	1.28 ± 0.21
Kinixys spekii	-0.86 ± 0.49	0.77 ± 0.32
Kinixys zombensis	-0.97 ± 0.18	1.31 ± 0.22
Kinosternon alamosae	-0.71 ± 0.51	0.60 ± 0.32
Kinosternon baurii	-0.31 ± 0.24	1.42 ± 0.21
Kinosternon chimalhuaca	-0.49 ± 0.70	0.12 ± 0.93
Kinosternon durangoense	-0.12 ± 0.11	0.66 ± 0.13
Kinosternon flavescens	-0.13 ± 0.14	0.63 ± 0.19
Kinosternon hirtipes	-0.86 ± 0.36	0.87 ± 0.29
Kinosternon integrum	-0.92 ± 0.11	0.49 ± 0.18
Kinosternon scorpioides	-0.38 ± 0.16	1.33 ± 0.35
Kinosternon sonoriense	-0.23 ± 0.14	0.82 ± 0.18
Kinosternon subrubrum	-0.21 ± 0.16	1.04 ± 0.20
Lepidochelys kempii	1.50 ± 0.11	-0.95 ± 0.18
Lepidochelys olivacea	1.49 ± 0.11	-0.92 ± 0.18
Leucocephalon yuwonoi	-1.47 ± 0.57	1.85 ± 0.36
Lissemys punctata	-0.68 ± 0.09	-0.02 ± 0.18

Lissemys scutata	-0.66 ± 0.12	-0.05 ± 0.22
Macrochelys temminckii	0.86 ± 0.10	0.08 ± 0.20
Malaclemys terrapin	-0.89 ± 0.06	-0.11 ± 0.12
Malacochersus tornieri	-1.26 ± 0.22	1.76 ± 0.21
Malayemys subtrijuga	-1.23 ± 0.09	0.52 ± 0.23
Manouria emys	1.70 ± 0.08	0.06 ± 0.19
Manouria impressa	0.54 ± 0.49	0.41 ± 0.30
Mauremys annamensis	-0.49 ± 0.48	0.24 ± 0.91
Mauremys caspica	-0.57 ± 0.10	-0.21 ± 0.22
Mauremys japonica	-0.89 ± 0.09	0.16 ± 0.21
Mauremys leprosa	-0.38 ± 0.08	0.11 ± 0.14
Mauremys mutica	-1.26 ± 0.18	1.44 ± 0.20
Mauremys nigricans	-1.09 ± 0.11	0.73 ± 0.21
Mauremys reevesii	-0.79 ± 0.14	0.82 ± 0.19
Mauremys rivulata	-0.41 ± 0.10	-0.54 ± 0.13
Mauremys sinensis	-0.75 ± 0.10	0.62 ± 0.19
Mecistops cataphractus	0.47 ± 0.09	0.25 ± 0.18
Melanochelys tricarinata	-1.25 ± 0.49	1.37 ± 0.25
Melanochelys trijuga	-0.35 ± 0.17	1.04 ± 0.26
Melanosuchus niger	-0.36 ± 0.11	-0.89 ± 0.18
Mesoclemmys dahli	-0.63 ± 0.12	0.78 ± 0.18
Mesoclemmys gibba	-2.06 ± 0.09	0.34 ± 0.31
Mesoclemmys nasuta	-1.17 ± 0.09	0.31 ± 0.20
Mesoclemmys tuberculata	-0.70 ± 0.53	0.30 ± 0.26
Mesoclemmys vanderhaegei	-0.72 ± 0.45	0.73 ± 0.28
Mesoclemmys zuliae	-0.67 ± 0.5	0.34 ± 0.25
Myuchelys bellii	-0.09 ± 0.1	-0.23 ± 0.23
Myuchelys georgesi	-0.06 ± 0.07	-0.31 ± 0.18
Myuchelys latisternum	-0.10 ± 0.09	-0.24 ± 0.21
Nilssonia gangetica	-0.53 ± 0.11	-0.75 ± 0.19
Notochelys platynota	-0.82 ± 0.43	0.34 ± 0.81
Orlitia borneensis	-0.48 ± 0.53	0.10 ± 0.29
Osteolaemus tetraspis	0.46 ± 0.12	0.69 ± 0.31
Palea steindachneri	0.14 ± 0.08	0.24 ± 0.12
Paleosuchus palpebrosus	-0.30 ± 0.06	-0.09 ± 0.12
Paleosuchus trigonatus	-0.02 ± 0.06	-0.06 ± 0.11
Pangshura smithii	-1.01 ± 0.08	0.06 ± 0.19
Pangshura sylhetensis	-0.49 ± 0.55	0.08 ± 0.32
Pangshura tecta	-1.18 ± 0.09	-0.10 ± 0.23
Pangshura tentoria	-1.26 ± 0.08	0.04 ± 0.20
Pelochelys bibroni	-0.99 ± 0.13	-0.59 ± 0.25
Pelodiscus sinensis	-1.33 ± 0.08	-0.65 ± 0.20
Pelomedusa subrufa	-0.35 ± 0.07	-0.36 ± 0.15
Pelusios adansonii	-1.97 ± 0.08	-0.40 ± 0.24
Pelusios bechuanicus	0.05 ± 0.53	-0.80 ± 0.27
Pelusios castaneus	0.38 ± 0.12	0.06 ± 0.19
Pelusios castanoides	0.52 ± 0.08	-0.22 ± 0.18
Pelusios chapini	0.22 ± 0.10	0.38 ± 0.19

Pelusios nanus	-0.58 ± 0.41	0.45 ± 0.27
Pelusios niger	-0.78 ± 0.09	-0.22 ± 0.20
Pelusios rhodesianus	0.33 ± 0.09	0.14 ± 0.18
Pelusios sinuatus	-1.03 ± 0.12	-0.97 ± 0.26
Pelusios subniger	0.19 ± 0.14	0.41 ± 0.23
Pelusios upembae	0.20 ± 0.44	-1.02 ± 0.20
Pelusios williamsi	-0.42 ± 0.49	-0.12 ± 0.31
Phrynops geoffroanus	0.09 ± 0.08	0.12 ± 0.16
Phrynops hilarii	0.27 ± 0.07	-0.16 ± 0.17
Phrynops williamsi	-0.53 ± 0.48	0.27 ± 0.30
Platemys platycephala	-1.51 ± 0.21	1.68 ± 0.23
Platysternon megacephalum	-0.84 ± 0.16	1.11 ± 0.29
Podocnemis erythrocephala	-0.76 ± 0.11	0.12 ± 0.18
Podocnemis expansa	1.12 ± 0.13	-1.12 ± 0.21
Podocnemis lewyana	-0.45 ± 0.50	-0.13 ± 0.26
Podocnemis sextuberculata	0.71 ± 0.08	-0.39 ± 0.19
Podocnemis unifilis	0.84 ± 0.08	0.07 ± 0.14
Podocnemis vogli	-0.29 ± 0.60	-0.15 ± 0.31
Psammobates geometricus	-0.55 ± 0.13	0.80 ± 0.16
Psammobates tentorius	-1.23 ± 0.46	1.50 ± 0.31
Pseudemydura umbrina	-0.77 ± 0.49	0.50 ± 0.30
Pseudemys alabamensis	-1.85 ± 0.11	-0.80 ± 0.25
Pseudemys concinna	0.33 ± 0.06	0.02 ± 0.16
Pseudemys gorzugi	0.26 ± 0.09	0.15 ± 0.19
Pseudemys nelsoni	-0.25 ± 0.07	-0.02 ± 0.12
Pseudemys peninsularis	-0.84 ± 0.07	-0.46 ± 0.11
Pseudemys rubriventris	-1.78 ± 0.12	-0.92 ± 0.24
Pseudemys texana	0.22 ± 0.07	0.23 ± 0.16
Pyxis arachnoides	-0.50 ± 0.56	2.11 ± 0.34
Pyxis planicauda	-0.43 ± 0.49	2.23 ± 0.35
Rheodytes leukops	-0.15 ± 0.47	-0.45 ± 0.28
Rhinemys rufipes	-0.49 ± 0.50	0.28 ± 0.26
Rhinoclemmys annulata	-1.29 ± 0.46	1.46 ± 0.26
Rhinoclemmys areolata	-1.48 ± 0.20	1.58 ± 0.23
Rhinoclemmys diademata	-2.31 ± 0.11	0.63 ± 0.29
Rhinoclemmys funerea	-0.93 ± 0.47	0.94 ± 0.22
Rhinoclemmys melanosterna	-2.37 ± 0.11	0.70 ± 0.25
Rhinoclemmys nasuta	-1.46 ± 0.56	1.88 ± 0.30
Rhinoclemmys pulcherrima	-1.09 ± 0.12	0.97 ± 0.25
Rhinoclemmys punctularia	-2.52 ± 0.12	0.95 ± 0.31
Rhinoclemmys rubida	-1.77 ± 0.17	1.08 ± 0.24
Sacalia bealei	-0.66 ± 0.12	0.81 ± 0.23
Sacalia quadriocellata	-1.00 ± 0.46	1.22 ± 0.26
Siebenrockiella crassicollis	-1.66 ± 0.22	1.62 ± 0.21
Staurotypus salvinii	-0.56 ± 0.12	0.54 ± 0.16
Staurotypus triporcatus	-0.15 ± 0.10	0.32 ± 0.14
Sternotherus carinatus	-0.56 ± 0.17	0.76 ± 0.17
Sternotherus depressus	-0.10 ± 0.21	1.43 ± 0.20

Sternotherus minor	-0.84 ± 0.17	0.86 ± 0.25
Sternotherus odoratus	-0.13 ± 0.21	1.27 ± 0.31
Stigmochelys pardalis	0.68 ± 0.13	1.05 ± 0.23
Terrapene carolina	0.83 ± 0.20	1.43 ± 0.26
Terrapene coahuila	-0.98 ± 0.13	0.58 ± 0.22
Terrapene nelsoni	-0.86 ± 0.15	0.97 ± 0.20
Terrapene ornata	0.33 ± 0.16	1.22 ± 0.16
Testudo graeca	0.71 ± 0.19	1.49 ± 0.20
Testudo kleinmanni	-0.69 ± 0.19	1.67 ± 0.18
Testudo marginata	-0.73 ± 0.08	0.43 ± 0.18
Tomistoma schlegelii	0.98 ± 0.07	0.08 ± 0.17
Trachemys callirostris	0.56 ± 0.12	0.71 ± 0.13
Trachemys decorata	-0.30 ± 0.51	0.01 ± 0.31
Trachemys decussata	-0.41 ± 0.09	0.53 ± 0.14
Trachemys dorbigni	0.57 ± 0.10	0.67 ± 0.18
Trachemys gaigeae	0.57 ± 0.11	0.64 ± 0.20
Trachemys grayi	0.54 ± 0.14	0.71 ± 0.25
Trachemys nebulosa	0.58 ± 0.11	0.69 ± 0.11
Trachemys ornata	0.51 ± 0.10	0.82 ± 0.14
Trachemys scripta	0.50 ± 0.11	0.79 ± 0.11
Trachemys stejnegeri	-1.48 ± 0.09	-0.19 ± 0.25
Trachemys taylori	0.57 ± 0.12	0.67 ± 0.13
Trachemys terrapen	-1.51 ± 0.11	0.76 ± 0.21
Trachemys venusta	0.53 ± 0.12	0.76 ± 0.16
Trachemys yaquia	0.57 ± 0.10	0.65 ± 0.17
Trionyx triunguis	0.76 ± 0.08	-0.46 ± 0.17
Vijayachelys silvatica	-1.07 ± 0.52	1.16 ± 0.35

Supplementary Figure

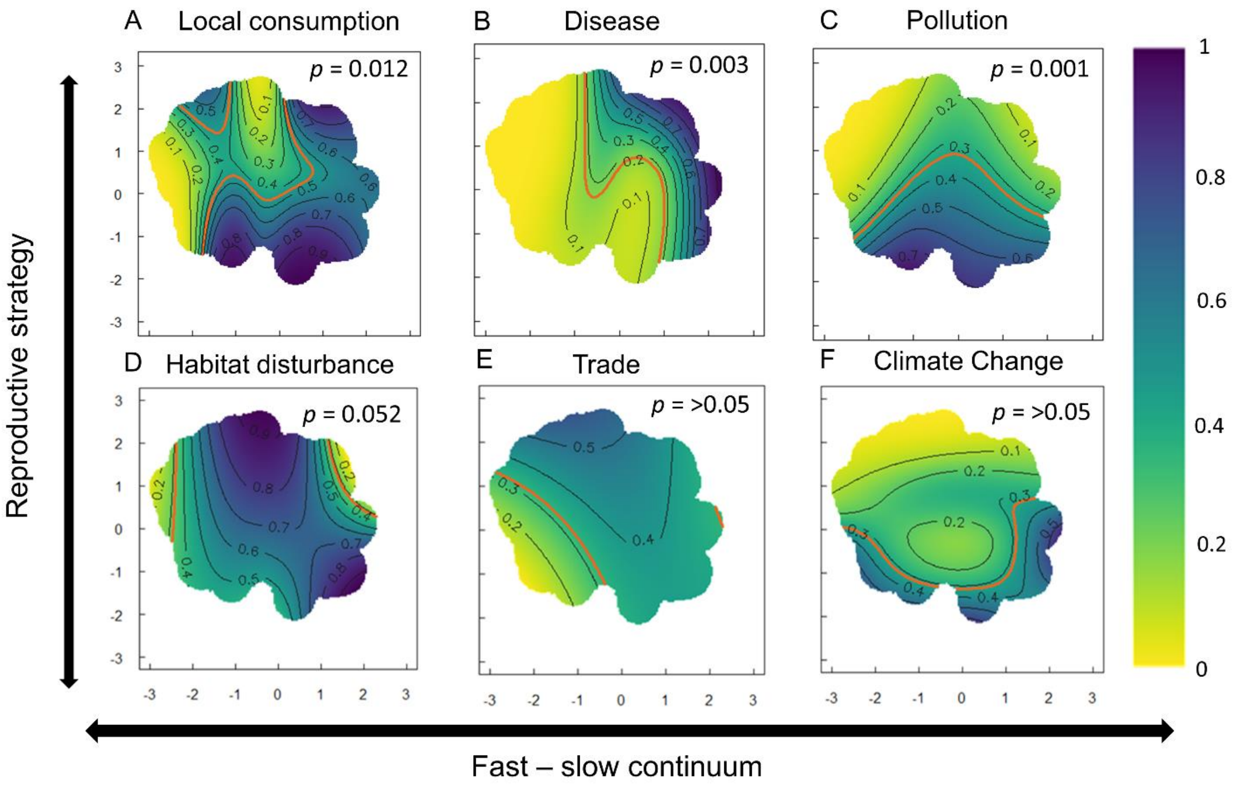

Supplementary Figure 1. The life history strategies of Testudines and Crocodilia predict the vulnerability to extinction due to some but not all considered threats. Probability of species being affected by (A) local consumption, (B) disease, (C) pollution, (D) habitat disturbances, (E) unsustainable global trade, and (F) climate change, according to GAMs (with binomial distribution) using the position of species in the two-dimensional functional space as predictors. Local consumption, disease, and pollution can predict the extinction of some life history strategies. Other risks, such as climate change, global trade, or habitat disturbance, do not show a significant relation with the species' life history strategie**s.** Yellow tones indicate lower risk of extinction due to each threat, whereas purple tones indicate higher risk. P values associated with each GAM are shown at the top-right corner of each panel, and $\chi 2$ is 28.79 for model A; 27.36 for B; 25.92 for C; 19.22 for D; 4.36 for E and 10.6 for F. The red contour lines indicate the average threat probability. We consider only species for which threats are known (n = 251 species).

Supplementary Methods

Data Selection

We drew life history data from the COMADRE Animal Matrix Database[2], DATLife Database[3], Amniote Life History Database[4], and the reviews by Allen et al.[5], Pfaller et al.[6] and Reinke et al.[7].

COMADRE is an open-access database that includes matrix population models (MPMs[8]) of 414 animal species around the globe[2]. MPMs describe the lifecycle transitions of individuals across discrete state classes (*i.e.*, age/stage/size classes). We selected MPMs of species in the orders Testudines and Crocodilia for which the MPMs: (i) were parameterised with field data from natural populations, *i.e.* undisturbed, unmanipulated populations, so their life history traits represent those of natural populations; (ii) encompass the entire life cycle of the species (*i.e.* include rates of survival, development, and reproduction along the full life cycle), so we could then calculate life history traits that require full life cycle information; (iii) have three or more life cycle stages, since lower-dimension MPMs lack the necessary resolution to estimate life history traits[9]; (iv) in the case of more than one study for the same species, the MPM with overall greater temporal replication was determined to better represent the species' demography. MPMs in COMADRE contain three sub-matrices necessary to quantify life history traits[8]: a state-specific survival sub-matrix ($\boldsymbol{U}$), a stage-specific per capita reproduction sub-matrix ($\boldsymbol{F}$), and a stage-specific per capita clonality sub-matrix ($\boldsymbol{C}$). The stage-classified population projection matrix is then defined as $\boldsymbol{A} = \boldsymbol{U} + \boldsymbol{F} + \boldsymbol{C}$. In our study species, $\boldsymbol{C} = (0)_{n \times n}$ (where n = life cycle stage number), as these taxonomic groups do not reproduce clonally. This set of criteria resulted in 24 MPMs for five Crocodilia species and 19 Testudines.

DATLife (Demography Across the Tree of Life) is an open-access database that includes life tables, longevity, and age at maturity for over 5,500 species[3]. The associated

life tables contain the age-specific survivorship (l_x) and age-specific fertility (m_x) in populations where individual age (x) is known. We selected the life tables of species in the order Testudines and Crocodilia that were parameterised strictly with field data from wild populations and, as for the COMADRE data, in the case of more than one study available for the same species, we selected the study with the longest temporal replication. We obtained life history trait data for 122 species of Crocodilia (14) and Testudines (108) from this resource.

The Amniote Life History Database (Amniote) includes life history information of 21,223 species of birds, mammals, and reptiles[4]. Amniote pulls information from empirical peer-reviewed studies on individual species, macroecological studies of multiple species, existing life history databases, published books, and other compilations. From Amniote, we obtained primarily reproductive life history traits (*e.g.*, age at maturity for males and females, clutch size, clutches per year, duration of gestation, duration of weaning, incubation time, egg mass, inter-clutch interval), survival-related life history traits (*e.g.*, longevity and maximum lifespan), as well as biometric information (*e.g.*, body mass, hatching weight, male and female body mass, straight ventral length for males, females and hatchings). Where multiple values are available for a species, Amniote reports the median value[4]. We obtained data from 237 species of Crocodilia (23) and Testudines (214) from this resource.

To complement the information about life history traits obtained from the aforementioned databases, we carried out a search of peer-reviewed studies that present demographic data for tortoises, turtles, crocodilians and alligators. Allen et al.[5], Pfaller et al.[6] and Reinke et al.[7] review and compile information about reproduction and survival of reptiles into open access databases. Allen et al.[5] collected life history data about reproduction and biometric information by combining existing life history databases and

supplementing these with additional data from the primary literature of 5,716 amphibian and 9,046 reptile species. In cases where Allen et al. [5] and the Amniote database reported different values for a species' traits, we selected the information of Allen et al. [5] for our analysis because it the data were more recent. Pfaller et al.[6] published a review compiling annual survival probability estimates through capture-recapture data for adult sea turtles. Reinke et al.[7] collected capture-recapture information to develop survival estimates for 45 reptile species.

To calculate the life history strategies of Testudines and Crocodilia, we collected/estimated the life history traits according to the different datasets:

- Mean number of clutches per year and clutch size (*CN* and *CS*, respectively) were obtained from Amniote[4] and Allen et al. [5]. Allen et al. [5] includes several data (including Amniote) and, when they found multiple records of the same trait for a species, the average of the species' trait was estimated by taking the mean of unique records per species. When the same species was present in both datasets, we selected the value in Allen et al. [5].
- Adult and juvenile survival (*Sa* and *Sj*, respectively) were calculated from the MPMs in COMADRE[2], life tables in DATLife[3], and from direct estimates of capture-recapture published studies[6,7]. Adult survival (*Sa*) from MPMs was estimated as the column sum of the matrix ***U*** of the stages representing reproductive individuals represented in the sub-matrix ***F*** (considering adults, all ages/stages after the first reproduction). We used arithmetic averages for the resulting stage-specific survival values, rather than weighting them by the stable stage distribution because we collected juvenile and adult data from other sources where calculating this weighted mean would not feasible, thus rendering comparisons impossible. In DATLife, we used age-specific fertility to determine the reproductive ages, and

then estimated adult survival as the arithmetic average of age-specific survival rates after the first reproduction. We estimated juvenile survival (*Sj*) using a similar approach: as the average of the sum of the columns that represent juveniles (all the stages prior to the first reproduction) in the pertinent sub-matrix ***U*** in COMADRE. In DATLife, we estimated the average of non-reproductive age- or stage-specific survival (prior to the first reproduction) of the life tables.

- Age at maturity (*Lα*) and maximum lifespan (*ML*) were estimated with the MPMs from COMADRE or obtained directly from the databases with this information available (such as Amniote or DATLife). In COMADRE, *Lα* was calculated using age-from-stage decompositions[8]. Briefly, we defined the reproductive stages as those columns of the ***F*** sub-matrix that contain values greater than zero. We estimated the mean time between birth and the first entry into the reproductive stage. This conditional mean is obtained by creating an absorbing state corresponding to the event of reproducing at least once before death, with a Markov chain, and calculating the mean time to absorption[8]. *ML* was estimated from the MPMs of COMADRE projecting 100 individuals in the first state and iterating up to 1000 years to identify the first year with fewer than one individual in the virtual cohort. We also used the databases Amniote and DATLife because they contain information about *Lα* and *ML* of most of the examined species. When several values from different datasets for one species and life history trait were available, we selected the lowest value for *Lα* and the highest values for *ML*. The rationale behind this choice is that the lowest value of *Lα* identifies the most likely first age at maturity reported in the life cycle of this species, whereas the highest values of *ML* approximate the maximum values of this trait reported for the species of interest in wildlife populations.

Missing data

For some species, specific life history traits were not directly available from databases/literature, nor were we able to calculate them with the demographic data that were available. This resulted in gaps in our dataset of species' life history traits. Thus, we explored the patterns of missing values for Testudines and Crocodilia species (Supplementary Figure 2). The traits with the highest proportion of missing values were juvenile survival (12.7% of all species) and adult survival (13.5%). Clutch number was available for 37.8% and age at maturity for 44%. The traits best represented in the database were maximum lifespan (79.9%) and clutch size (97.7%). Overall, the percentage of gaps was 38%, then we imputed around 1 dataset for every percent missing, so we imputed 40 datasets.

We also find a weak trend between the conservation status and the number of missing traits (Supplementary Figure 3). This relation is significant (Kruskal Wallis test: P = 0.003, $\chi 2$ = 8.992, df = 1), but rather weak: the mean number of missing traits for threatened species was 3.08±1.50, while the mean of missing traits for non-threatened species was 2.42±1.54.

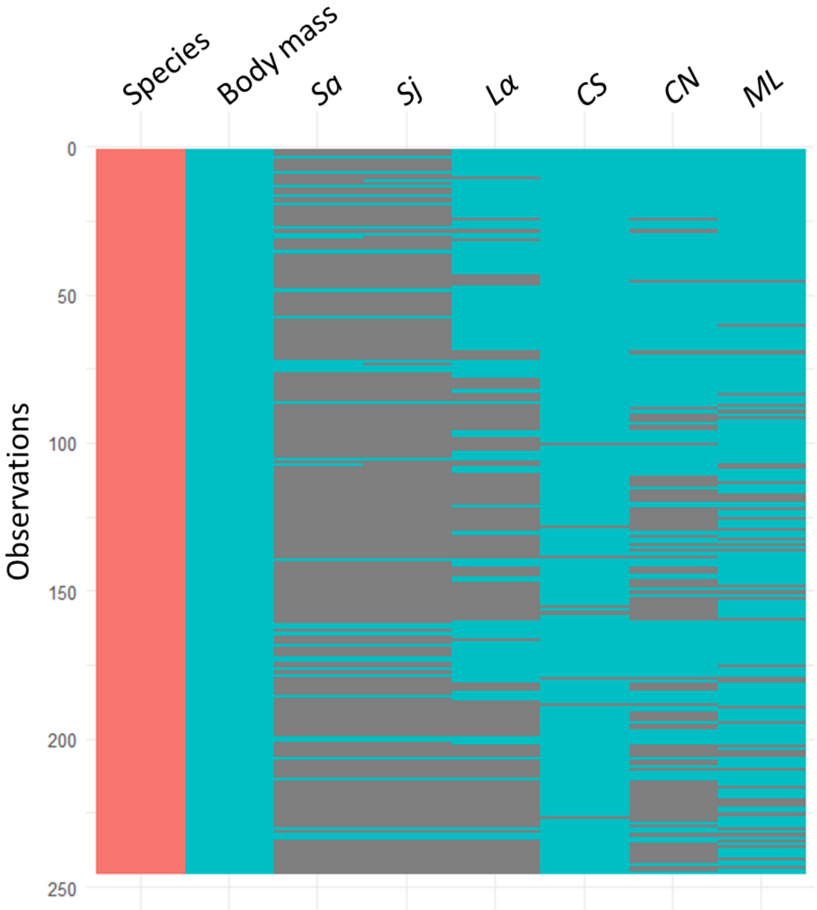

Supplementary Figure 2. Proportion of missing life history trait values for the studied species. Despite our search of literature and existing datasets, we were unable to find values for all life history traits for all species (*N* = 256). The life history traits are: adult survival (Sa), juvenile survival (Sj), maximum lifespan (ML), age at sexual maturity (Lα), mean of number of clutches per year (CN), clutch size (CS). Red values are character records such as the name of the species, blue values are numeric information and grey values are gaps of information (NA data).

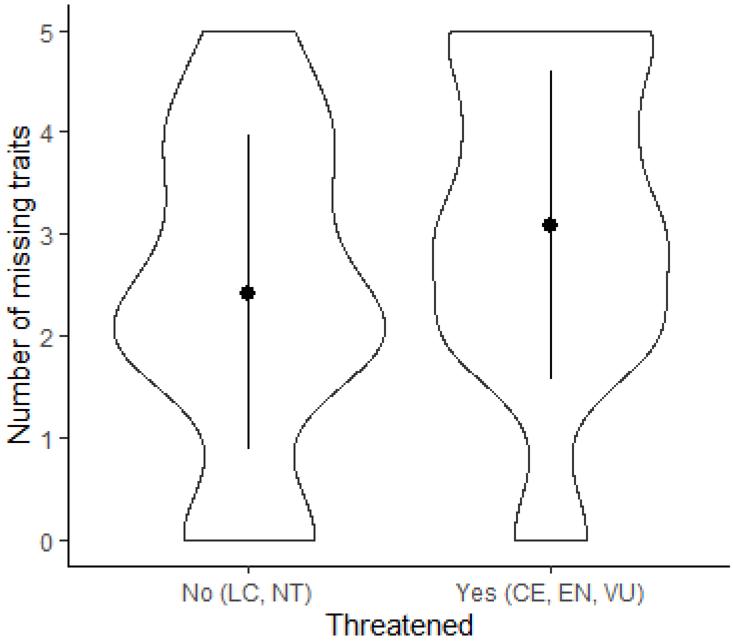

Supplementary Figure 3. Threatened species in our study have more missing life history traits (mean 3.08 missing traits) than non-threatened species (mean 2.42 missing traits). The threatened conservation status here is collapsed into two groups according to the IUCN Red List: "No" (Least Concerns and Near Threatened) and "Yes" (Vulnerable, Endangered, and Critical Endangered). Boxes represent the interquartile range, the horizontal line represents the median, vertical line represents the upper and lower extreme values of the 95% of the interquartile range. Even the difference is very weak, we found a significant difference (Kruskal Wallis test: $P = 0.003$, $\chi^2 = 8.992$, $df = 1$).

Imputation validation

Because the *p*PCA analyses require complete datasets, we imputed the missing data using the add-on *phylomice* to *Mice* package[10], as detailed in the Methods section *Phylogeny and data imputation* of our main manuscript.

To evaluate the collinearity between traits, we examined the pair-wise correlations of traits before and after the imputation analyses (Supplementary Table 3 and 4, respectively). The correlation between traits is rather low, and it becomes even lower in the case of the imputed data. Age at maturity (*Lα*) and maximum longevity (*ML*) showed the higher correlation with the original data before the imputation.

Supplementary Table 3. Correlation matrix between life history traits using the un-imputed data of our 259 species of Testudines and Crocodilia. Note that none of the pairwise correlations are strong, with the highest value at <0.5. The life history traits are: adult survival (*Sa*), juvenile survival (*Sj*), maximum lifespan (*ML*), age at sexual maturity (*Lα*), mean of number of clutches per year (*CN*), clutch size (*CS*).

	Sj	*Lα*	*CS*	*CN*	*ML*
Sa	0.013	0.313	-0.060	0.082	0.180
Sj		0.341	0.038	0.049	-0.086
Lα			0.031	0.231	0.493
CS				-0.357	0.246
CN					0.162

Supplementary Table 4. Correlation matrix between life history traits using the imputed data of our 259 species of Testudines and Crocodilia. The standard deviation (SD) is included for each correlation using the 40 imputed datasets. Note that none of the pairwise correlations are strong, with the highest value at <0.5. The life history traits are: adult survival (*Sa*), juvenile survival (*Sj*), maximum lifespan (*ML*), age at sexual maturity (*Lα*), mean of number of clutches per year (*CN*), clutch size (*CS*).

	Sj	*Lα*	*CS*	*CN*	*ML*
Sa	0.057±0.06	0.105±0.07	0.065±0.06	0.043±0.06	0.081±0.07
Sj		0.083±0.07	0.112±0.07	0.024±0.06	0.085±0.06
Lα			0.185±0.05	0.075±0.07	0.188±0.09
CS				-0.079±0.02	0.194±0.02
CN					0.121±0.03

We compared the distribution of the imputed data versus original data (with no missing values) using density plots (Supplementary Figure 4). Visually we can identify that the data from the 40 imputed datasets matched with the values with the original data.

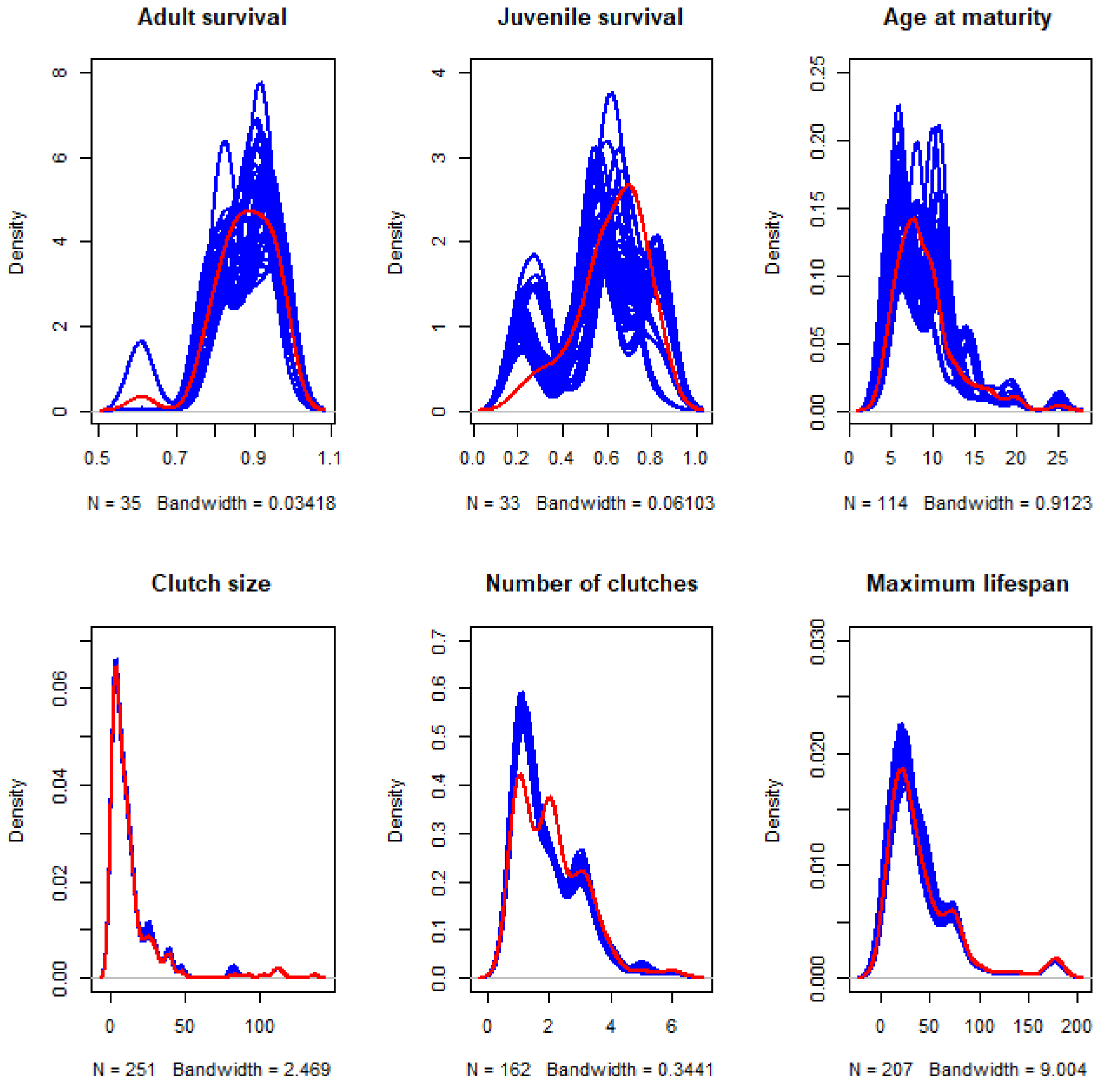

Supplementary Figure 4. Comparison density plots between imputed *versus* non-imputed data for each life history trait. In red, the density of values of observed data, in blue the datasets estimated in each imputation (including observed and imputed values). *N* is the number of observed values per trait, for example, in adult survival we have information available for 35 species and 251 species for clutch size. Bandwidth was fixed according to the observed data in the density plot, in other words, the bandwidth used in the red line (observed data) was the same that it was used for all imputations.

To evaluate the performance of the imputation method utilised in our study, we compared the main results of the *p*PCA with imputed data versus a subset of the dataset where a maximum of two traits were imputed. This new subset encompassed 108 species and we have no gaps for *CS* and *Lα,* the percentage of gap information for *CN* was 0.9 %, for *ML* was 1.9%. However, the data with the highest empty values are those related to survival (*Sa* = 68.5% and *Sj* = 69.4%.). We performed a *p*PCA using this subset of 108 species. Visually we could see that the main trends of the analyses are similar (Supplementary Figure 5). The Pagel's λ of the new model with few imputed data was 0.825, slightly higher than the model with imputed data. The proportion of variance explained by the *pPCA* was also higher for the first axis, PC1 = 42.39%, and it was similar for the second axis, PC2 = 25.28%. This similarity in the results support the use of the imputed data.

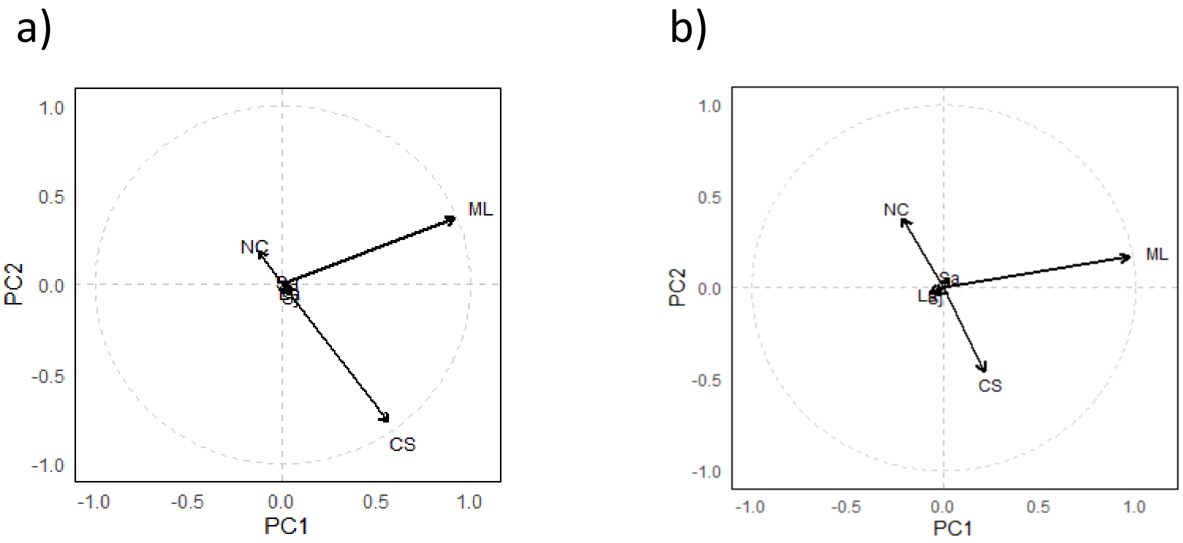

Supplementary Figure 5. Similar loadings of *pPCA* corrected by size are shown in the two models: a) using imputed data to fill gaps, so that all species could be included (N = 259); and b) using only species with a maximum of two imputed traits (N = 108). Representation of the axis of variation for the model used in the manuscript (a) and the model with a selection of species without imputed data (b).

To quantify the differences between both *p*PCA analyses, we did a Procrustes analysis. This analysis shows up to what point the position of the common species (108 species) remains constant between the two spaces. To carry on these analyses, we used the function procrustes from the *vegan* package[11] in R. The results showed high correlation in a symmetric Procrustes rotation (0.9656) between both datasets and the sum of squared distances between paired points in the ordination space was 0.067 (Supplementary Figure 6). The permutational test of the significance of the Procrustes was done with 9999 permutations, and showed significant relations between the scores of imputed database and scores where a maximum of two traits were imputed ($p < 0.001$). This result means high correlation between both *p*PCA analyses, supporting the use of the imputed data in the analyses.

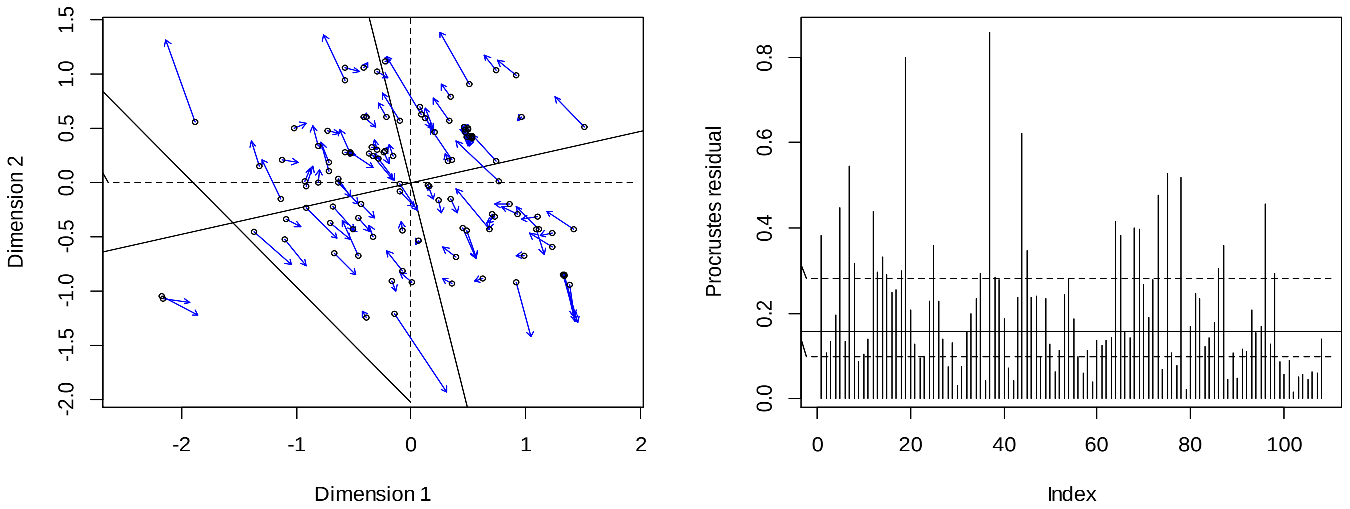

Supplementary Figure 6. Representation of the Procrustes errors. On the left, visual indication of the degree of match between the two spaces. Symbols show the position of the samples in the *p*PCA scores with a maximum of two traits imputed per species, and arrows point to their positions according to the score of the *p*PCA with the imputed data. The plot also shows the axis rotation between the two ordinations. On the right, the plot shows the residuals for each species. The horizontal lines, from bottom to top, are the 25% (dashed), 50% (solid), and 75% (dashed) quantiles of the residuals.

Body mass data

Body mass is highly correlated with many life history traits[12,13], and therefore in this manuscript our multivariate analyses account for body mass. We collected the female body mass values for all studied species using the datasets described in the section *Data selection* of the Supplementary Methods. We also searched for body mass data in the literature, including data presented under previously taxonomically accepted names for a given species. When we were not able to find a value for body mass, we estimated body mass using the total length of the species (using the information of the same clade, *i.e.* family or genus). We conducted simple regression between body mass and total length because these traits are highly correlated, we used all the species corresponding to the taxonomic genus or family, according to the number of species available in each taxonomic group. We estimated body mass using this approach for 10 of the 259 species included in the analyses (Supplementary Table 5).

Supplementary Table 5. List of species used in the manuscript for which females body mass (g.) was available.

Order	Family	Species	BM	Database
Crocodilia	Alligatoridae	*Alligator mississippiensis*	47800	Amniote Database[4]
		Alligator sinensis	10750	Amniote Database[4]
		Caiman crocodilus	10550	Amniote Database[4]
		Caiman latirostris	14600	Amniote Database[4]
		Caiman yacare	10550	Amniote Database[4]
		Melanosuchus niger	82000	Amniote Database[4]
		Paleosuchus palpebrosus	5900	Amniote Database[4]
		Paleosuchus trigonatus	7500	Amniote Database[4]
	Crocodylidae	*Crocodylus acutus*	76700	Amniote Database[4]
		Crocodylus intermedius	107900	Amniote Database[4]
		Crocodylus johnsoni	19500	Amniote Database[4]
		Crocodylus mindorensis	38400	Amniote Database[4]
		Crocodylus moreletii	31700	Amniote Database[4]
		Crocodylus niloticus	94200	Amniote Database[4]
		Crocodylus novaeguineae	39900	Amniote Database[4]

		Crocodylus palustris	42700	Amniote Database[4]
		Crocodylus porosus	78700	Amniote Database[4]
		Crocodylus rhombifer	57500	Amniote Database[4]
		Crocodylus siamensis	42500	Amniote Database[4]
		Mecistops cataphractus	50500	Amniote Database[4]
		Osteolaemus tetraspis	18800	Amniote Database[4]
		Tomistoma schlegelii	119000	Amniote Database[4]
	Gavialidae	*Gavialis gangeticus*	147000	Allen et al. [5]
Testudines	Carettochelyidae	*Carettochelys insculpta*	10050	Amniote Database[4]
	Chelidae	*Acanthochelys pallidipectoris*	369.2	Amniote Database[4]
		Acanthochelys spixii	330.87	Fraxe Neto et al. [14]
		Chelodina longicollis	1293	Amniote Database[4]
		Chelodina mccordi	2340	Colston et al. [15]
		Chelodina novaeguineae	1006	Amniote Database[4]
		Chelodina reimanni	1006	Amniote Database[4]
		Elseya dentata	4295	Amniote Database[4]
		Emydura macquarii	1740	Amniote Database[4]
		Emydura subglobosa	1030	Amniote Database[4]
		Emydura tanybaraga	1030	Amniote Database[4]
		Hydromedusa maximiliani	325	Regis and Meik [16]
		Hydromedusa tectifera	1155	Regis and Meik [16]
		Mesoclemmys dahli	777	Amniote Database[4]
		Mesoclemmys gibba	706.8	Amniote Database[4]
		Mesoclemmys nasuta	2180	Cunha et al. [17]
		Mesoclemmys tuberculata	657.4	Regis and Meik [16]
		Mesoclemmys vanderhaegei	453.29	Regis and Meik [16]
		Mesoclemmys zuliae	1983	Amniote Database[4]
		Myuchelys bellii	1628	Fielder et al. [18]
		Myuchelys georgesi	977.3	Regis and Meik [16]
		Myuchelys latisternum	1377	Amniote Database[4]
		Phrynops geoffroanus	2233.3	Regis and Meik [16]
		Phrynops hilarii	2380	Amniote Database[4]
		Phrynops williamsi	4740.97	Encyclopedia of Life [19]
		Platemys platycephala	325	Amniote Database[4]
		Pseudemydura umbrina	324.1	Amniote Database[4]
		Rheodytes leukops	1691	Amniote Database[4]
		Rhinemys rufipes	1265.96	Estimated
	Cheloniidae	*Caretta caretta*	92400	Amniote Database[4]
		Chelonia mydas	128100	Amniote Database[4]
		Eretmochelys imbricata	62300	Amniote Database[4]
		Lepidochelys kempii	92400	Amniote Database[4]
		Lepidochelys olivacea	116300	Amniote Database[4]
		Chelydra rossignonii	3875	Amniote Database[4]
		Chelydra serpentina	3875	Amniote Database[4]
		Macrochelys temminckii	23500	Amniote Database[4]

Dermatemydidae	*Dermatemys mawii*	15660	Amniote Database[4]
Dermochelyidae	*Dermochelys coriacea*	372500	Amniote Database[4]
Emydidae	*Chrysemys dorsalis*	378	Amniote Database[4]
	Chrysemys picta	378	Amniote Database[4]
	Clemmys guttata	165.4	Amniote Database[4]
	Deirochelys reticularia	910.5	Amniote Database[4]
	Emys orbicularis	1096.49	Allen et al. [5]
	Glyptemys insculpta	968.2	Amniote Database[4]
	Glyptemys muhlenbergii	132.1	Amniote Database[4]
	Graptemys barbouri	1256	Amniote Database[4]
	Graptemys caglei	1085.91	Estimated
	Graptemys ernsti	1519	Aprox.[a]
	Graptemys flavimaculata	1130	Amniote Database[4]
	Graptemys geographica	1138	Amniote Database[4]
	Graptemys gibbonsi	2334.5	Amniote Database[4]
	Graptemys nigrinoda	415.7	Amniote Database[4]
	Graptemys oculifera	918.15	Amniote Database[4]
	Graptemys ouachitensis	1306.5	Amniote Database[4]
	Graptemys pearlensis	1530.26	Estimated
	Graptemys pseudogeographica	1477	Amniote Database[4]
	Graptemys pulchra	1519	Amniote Database[4]
	Graptemys versa	1477	Amniote Database[4]
	Malaclemys terrapin	886	Amniote Database[4]
	Pseudemys alabamensis	3238.5	Amniote Database[4]
	Pseudemys concinna	2992	Amniote Database[4]
	Pseudemys gorzugi	2992	Amniote Database[4]
	Pseudemys nelsoni	3738.5	Amniote Database[4]
	Pseudemys peninsularis	3065.5	Amniote Database[4]
	Pseudemys rubriventris	3238.5	Amniote Database[4]
	Pseudemys texana	2992	Amniote Database[4]
	Terrapene carolina	372	Amniote Database[4]
	Terrapene coahuila	259.8	Amniote Database[4]
	Terrapene nelsoni	372	Amniote Database[4]
	Terrapene ornata	391	Amniote Database[4]
	Trachemys callirostris	1854	Amniote Database[4]
	Trachemys decorata	2522	Amniote Database[4]
	Trachemys decussata	630.1	Amniote Database[4]
	Trachemys dorbigni	1854	Amniote Database[4]
	Trachemys gaigeae	1854	Amniote Database[4]
	Trachemys grayi	1813	Amniote Database[4]
	Trachemys nebulosa	1854	Amniote Database[4]
	Trachemys ornata	1813	Amniote Database[4]
	Trachemys scripta	1813	Amniote Database[4]
	Trachemys stejnegeri	2012.95	Encyclopedia of Life[19]
	Trachemys taylori	1854	Amniote Database[4]

	Trachemys terrapen	4364.33	Encyclopedia of Life[19]
	Trachemys venusta	1813	Amniote Database[4]
	Trachemys yaquia	1854	Amniote Database[4]
Geoemydidae	*Batagur baska*	17900	Amniote Database[4]
	Batagur borneoensis	16900	Amniote Database[4]
	Batagur dhongoka	7270	Amniote Database[4]
	Batagur kachuga	21100	Amniote Database[4]
	Batagur trivittata	21611.55	Encyclopedia of Life [19]
	Cuora amboinensis	1000	Amniote Database[4]
	Cuora aurocapitata	530.85	Estimated
	Cuora bourreti	499	Amniote Database[4]
	Cuora flavomarginata	499	Amniote Database[4]
	Cuora galbinifrons	1200	Colston et al. [15]
	Cuora mccordi	375	Amniote Database[4]
	Cuora mouhotii	850	Colston et al. [15]
	Cuora picturata	1100	Colston et al. [15]
	Cuora trifasciata	3000	Aprox.[b]
	Cuora yunnanensis	850	Amniote Database[4]
	Cyclemys dentata	1250	Amniote Database[4]
	Geoclemys hamiltonii	6000	Regis and Meik [16]
	Geoemyda japonica	196	Amniote Database[4]
	Geoemyda spengleri	196	Amniote Database[4]
	Hardella thurjii	10500	Regis and Meik [16]
	Heosemys annandalii	4000	Andersen et al. [20]
	Heosemys grandis	4600.78	Regis and Meik [16]
	Heosemys spinosa	950	Amniote Database[4]
	Leucocephalon yuwonoi	1152.27	Regis and Meik [16]
	Malayemys subtrijuga	3535	Allen et al. [5]
	Mauremys annamensis	1717	McCormack et al. [21]
	Mauremys caspica	912.01	Allen et al. [5]
	Mauremys japonica	494.2	Amniote Database[4]
	Mauremys leprosa	608	Amniote Database[4]
	Mauremys mutica	319.5	Amniote Database[4]
	Mauremys nigricans	912	Regis and Meik [16]
	Mauremys reevesii	858	Amniote Database[4]
	Mauremys rivulata	257.5	Regis and Meik [16]
	Mauremys sinensis	1241	Amniote Database[4]
	Melanochelys tricarinata	1800	Regis and Meik [16]
	Melanochelys trijuga	760	Amniote Database[4]
	Notochelys platynota	4364.33	Encyclopedia of Life[19]
	Orlitia borneensis	12400	Amniote Database[4]
	Pangshura smithii	919	Amniote Database[4]
	Pangshura sylhetensis	1150	Regis and Meik [16]
	Pangshura tecta	1000	Regis and Meik [16]
	Pangshura tentoria	1184	Amniote Database[4]

	Rhinoclemmys annulata	638.64	Estimated
	Rhinoclemmys areolata	731.5	Amniote Database[4]
	Rhinoclemmys diademata	1126	Amniote Database[4]
	Rhinoclemmys funerea	946	Amniote Database[4]
	Rhinoclemmys melanosterna	2700	Amniote Database[4]
	Rhinoclemmys nasuta	1284	Amniote Database[4]
	Rhinoclemmys pulcherrima	944.6	Regis and Meik [16]
	Rhinoclemmys punctularia	2344	Encyclopedia of Life [19]
	Rhinoclemmys rubida	287	Butterfield et al. [22]
	Sacalia bealei	329.6	Lin et al. [23]
	Sacalia quadriocellata	284.95	Regis and Meik [16]
	Siebenrockiella crassicollis	940	Amniote Database[4]
	Vijayachelys silvatica	230	Amniote Database[4]
Kinosternidae	*Claudius angustatus*	200	Amniote Database[4]
	Kinosternon alamosae	145	Amniote Database[4]
	Kinosternon baurii	143	Amniote Database[4]
	Kinosternon chimalhuaca	266	López-Luna et al. [24 c]
	Kinosternon durangoense	271.3	Amniote Database[4]
	Kinosternon flavescens	271.3	Amniote Database[4]
	Kinosternon hirtipes	202.6	Amniote Database[4]
	Kinosternon integrum	474.4	Amniote Database[4]
	Kinosternon scorpioides	266	Amniote Database[4]
	Kinosternon sonoriense	326	Amniote Database[4]
	Kinosternon subrubrum	152.35	Amniote Database[4]
	Staurotypus salvinii	900	Amniote Database[4]
	Staurotypus triporcatus	4200	Amniote Database[4]
	Sternotherus carinatus	248	Amniote Database[4]
	Sternotherus depressus	144	Amniote Database[4]
	Sternotherus minor	154.5	Amniote Database[4]
	Sternotherus odoratus	137.9	Amniote Database[4]
Pelomedusidae	*Pelomedusa subrufa*	2273	Amniote Database[4]
	Pelusios adansonii	1620	Regis and Meik [16]
	Pelusios bechuanicus	4740.97	Encyclopedia of Life [19]
	Pelusios castaneus	368.5	Rawski and Józefiak [25]
	Pelusios castanoides	800	Amniote Database[4]
	Pelusios chapini	3515.1	Estimated
	Pelusios nanus	311.93	Encyclopedia of Life [19]
	Pelusios niger	1510	Akani et al. [25]
	Pelusios rhodesianus	900	Amniote Database[4]
	Pelusios sinuatus	7000	Regis and Meik [16]
	Pelusios subniger	1232.64	Encyclopedia of Life [19]
	Pelusios upembae	1210.35	Estimated
	Pelusios williamsi	2246.59	Encyclopedia of Life [19]
Platysternidae	*Platysternon megacephalum*	305.5	Regis and Meik [16]
Podocnemididae	*Erymnochelys madagascariensis*	4900	Regis and Meik [16]

	Podocnemis erythrocephala	1412	Regis and Meik [16]
	Podocnemis expansa	25800	Amniote Database[4]
	Podocnemis lewyana	9599	Amniote Database[4]
	Podocnemis sextuberculata	25800	Amniote Database[4]
	Podocnemis unifilis	6380	Amniote Database[4]
	Podocnemis vogli	2013	Amniote Database[4]
Testudinidae	*Aldabrachelys gigantea*	33000	Amniote Database[4]
	Astrochelys radiata	7955	Amniote Database[4]
	Astrochelys yniphora	8800	Amniote Database[4]
	Chelonoidis becki	43360.3	Estimated
	Chelonoidis carbonarius	6087.5	Regis and Meik [16]
	Chelonoidis chathamensis	29954.54	Estimated
	Chelonoidis chilensis	3181	Amniote Database[4]
	Chelonoidis darwini	36238.49	Estimated
	Chelonoidis denticulatus	3675	Regis and Meik [16]
	Chelonoidis duncanensis	22000	Chiari et al. [27]
	Chelonoidis hoodensis	28000	Chiari et al. [27]
	Chelonoidis porteri	72000	Chiari et al. [27]
	Chelonoidis vicina	61000	Chiari et al. [27]
	Chersina angulata	715	Amniote Database[4]
	Geochelone elegans	2500	Amniote Database[4]
	Geochelone platynota	2500	Amniote Database[4]
	Gopherus agassizii	2443	Amniote Database[4]
	Gopherus berlandieri	1769.5	Amniote Database[4]
	Gopherus flavomarginatus	85000	McDonald [28]
	Gopherus polyphemus	2784	Amniote Database[4]
	Homopus areolatus	289.4	Amniote Database[4]
	Homopus femoralis	599	Amniote Database[4]
	Indotestudo elongata	255	Amniote Database[4]
	Indotestudo forstenii	967.5	Amniote Database[4]
	Indotestudo travancorica	255	Amniote Database[4]
	Kinixys belliana	1202	Amniote Database[4]
	Kinixys erosa	958.4	Regis and Meik [16]
	Kinixys homeana	690.7	Regis and Meik [16]
	Kinixys lobatsiana	1202	Amniote Database[4]
	Kinixys natalensis	1202	Amniote Database[4]
	Kinixys nogueyi	1202	Amniote Database[4]
	Kinixys spekii	617	Hailey and Coulson [29]
	Kinixys zombensis	1202	Amniote Database[4]
	Malacochersus tornieri	400	Amniote Database[4]
	Manouria emys	30000	Bonin et al. [30]
	Manouria impressa	3200	Amniote Database[4]
	Psammobates geometricus	366.8	Amniote Database[4]
	Psammobates tentorius	423	Amniote Database[4]
	Pyxis arachnoides	398.1	Regis and Meik [16]

	Pyxis planicauda	420	Regis and Meik [16]
	Stigmochelys pardalis	20000	Amniote Database[4]
	Testudo graeca	1430	Amniote Database[4]
	Testudo kleinmanni	295	Regis and Meik [16]
	Testudo marginata	2080	Amniote Database[4]
Trionychidae	*Amyda cartilaginea*	2500	Andersen et al. [20]
	Apalone ferox	20000	Amniote Database[4]
	Apalone mutica	819	Amniote Database[4]
	Apalone spinifera	4765	Amniote Database[4]
	Chitra indica	108000	Amniote Database[4]
	Cyclanorbis senegalensis	11300	Gramentz [31 d]
	Cycloderma frenatum	14591	Amniote Database[4]
	Lissemys punctata	1444.5	Amniote Database[4]
	Lissemys scutata	1444.5	Amniote Database[4]
	Nilssonia gangetica	19000	Amniote Database[4]
	Palea steindachneri	10100	Regis and Meik [16]
	Pelochelys bibroni	120000	Bonin et al. [30]
	Pelodiscus sinensis	2327.5	Amniote Database[4]
	Trionyx triunguis	10818	Amniote Database[4]

a. Previously *G. puchra* was considered a subspecies and both reach similar sizes.
b. *C. ciclormata* was consided the same species.
c. According to López-Luna et al. [24], the morphology is similar to *K. scorpiodes*.
d. Data of. *C. elegans*, which reach similar size

To examine the sensitivity of our results to our chosen approach of life history strategies, we repeated the *p*PCA analysis including the body mass as a trait, rather than correcting for it (Supplementary Table 6, Supplementary Figure 7). The results of this PCA support the general findings reported in the main paper. In this PCA, body mass loads strongly onto PC1, which absorbs 54.5% of the variance. PC2 represents the fast-slow continuum, showing a strongly relation with the maximum longevity (*ML*), and retains 17.4%. Finally, PC3 is linked to the reproductive strategy, highly related with clutch size, and with 9.11% of the explained variance. Our original approach, depicted in the main body of this work, where body mass was corrected for, shows qualitatively similar results, with the fast-slow continuum being represented in PC1 and the reproductive strategies continuum in PC2, explaining 31.1% and 23.1% of the variance respectively (See Figure 1/Supplementary Table 1). In other words, the loadings of PC2

and PC3 in the *p*PCA match the loadings of PC1 and PC2 in the *p*PCA corrected by body mass.

Supplementary Table 6. Loadings of phylogenetically-corrected principal component analysis (*p*PCA), including body mass as a trait, for the 236 species of Testudines and 23 species of Crocodilia examined in this study. Only the first three principal component (PC) axes are shown. Bold numbers indicate loading absolute values >0.50. Outputs correspond to the mean values of 40 imputed data sets. Pagel's λ indicates the extent to which patterns are explained (=1) or not (=0) by phylogenetic relationships. Variance estimates (±) correspond to standard deviation.

Life history trait	Symbol	PC1	PC2	PC3
Body mass	*BM*	**0.97 ± 0.00**	0.18 ± 0.02	0.14 ± 0.04
Adult survival	*Sa*	0.06 ± 0.04	-0.01 ± 0.06	0.03 ± 0.07
Juvenile survival	*Sj*	0.02 ± 0.06	-0.05 ± 0.10	-0.18 ± 0.50
Age at sexual maturity	*Lα*	0.16 ± 0.05	-0.01 ± 0.09	-0.06 ± 0.15
Clutch size	*CS*	**0.76 ± 0.01**	-0.08 ± 0.05	**-0.51 ± 0.13**
Mean number of clutches per year	*CN*	0.05 ± 0.03	0.04 ± 0.06	0.19 ± 24
Maximum lifespan	*ML*	0.29 ± 0.03	**-0.95 ± 0.01**	0.10 ± 0.04
**Proportion of variance explained**		54.4% ± 1.06%	17.4% ± 1.25%	9.11% ± 0.60%
**Cumulative proportion of variance explained**		54.4%	71.8%	80.90%
**Pagel's λ**			0.758 ± 0.018	

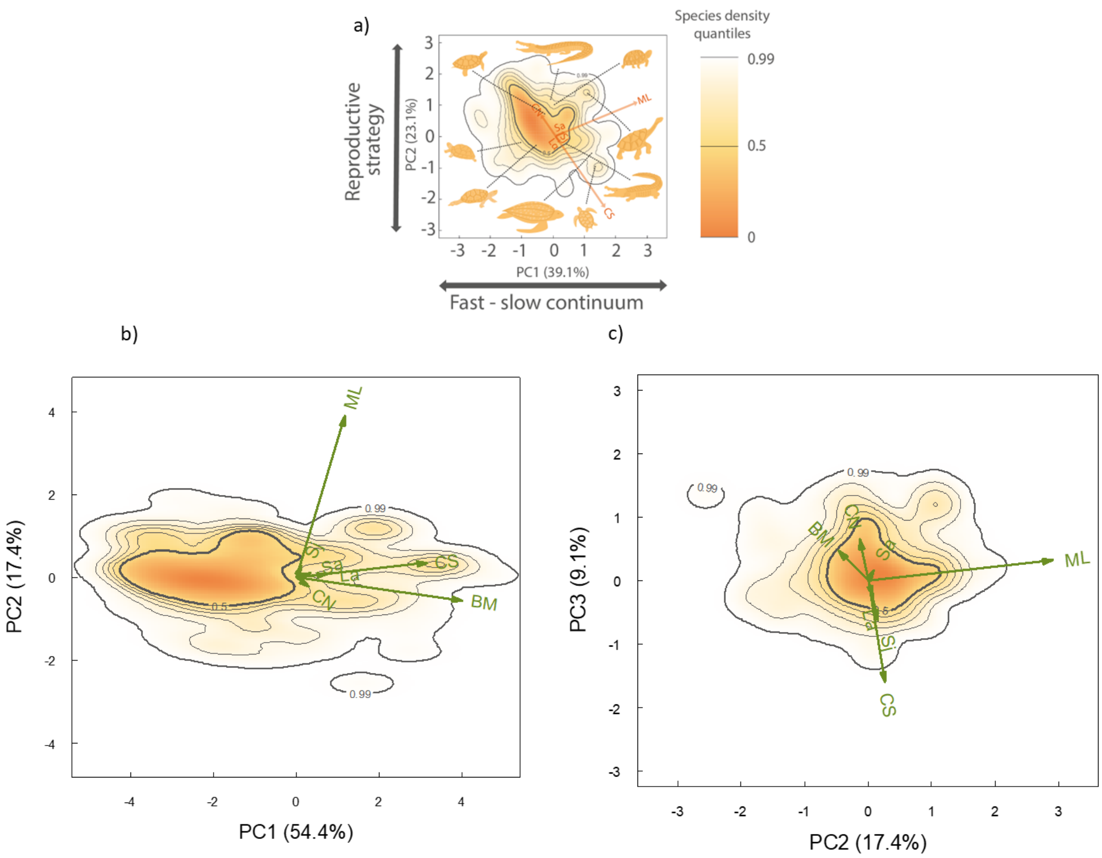

Supplementary Figure 7. a) The functional spectra of the life history strategies of Testudines and Crocodilia described in the main body of the manuscript using phylogenetically-corrected principal component analysis (*p*PCA) corrected by body mass. b) Representation of the spectra using the *p*PCA where body mass is included as a trait, together with the six examined life history traits, representation of PC1 (allometric continuum) and PC2 (fast-slow continuum). c) Representation of the spectra using the *p*PCA where body mass is included as a trait, representation of PC2 (fast-slow continuum) and PC3 (reproductive strategy). The phylogenetic signal is Pagel's λ = 0.674 ± 0.030 (SE) for a) and 0.758 ± 0.018 for b) and c). The life history traits are: adult survival (*Sa*), juvenile survival (*Sj*), maximum lifespan (*ML*), age at sexual maturity (*Lα*), mean of number of clutches per year (*CN*), clutch size (*CS*). Arrows indicate the direction and weighting of each trait in the pPCA. The colour gradient (orange, yellow, and white) depicts the density of species in the defined space, where orange corresponds to more densely populated areas). Thick contour lines indicate the 0.5 and 0.99 quantiles, and thinner ones indicate quantiles 0.6, 0.7, 0.8, and 0.9.

Phylogenetic signal

To explore the phylogenetic signal of each trait, we estimated Pagel's λ for each trait separately. This approach describes the strength of phylogenetic relationships on trait evolution under a Brownian motion model[32]. Pagel's λ ranges between 0, which indicates that the patterns in the traits cannot be explained by the employed phylogeny, and 1, which indicates that the observed patterns in traits are tightly correlated with the placement of species in the phylogeny. We used the function *phylosig* in the package *Phytools* [33] to estimate the phylogenetic signal of each trait before the imputation analyses (Supplementary Table 7). We estimated the phylogenetic signal for the original data and for the full dataset obtained via the phylogenetic imputation methods (Supplementary Table 7). The results show that the imputation did not alter the phylogenetic signal, as there is an overlap in the confidence intervals in all the traits before and after imputation.

Supplementary Table 7. Phylogenetic signal of the life history traits in Testudines and Crocodilia. Pagel's λ describes the statistical dependence among species' trait values due to their phylogenetic relationships, ranging between 1 (meaning a life history trait is fully related to the phylogenetic structure as explained by Brownian motion), and 0 (meaning no phylogenetic structuring of the trait). Pagel's λ was calculated for all traits included in the analyses. N represents the number of species of Testudines and Crocodilia with data for each trait. Pagel's λ was estimated for the traits after and before the imputation. The variance for imputed data was calculated with the standard deviation of the 40 datasets.

Life history trait	Symbol	N	Pagel's λ original data		Pagel's λ imputed data	
Adult survival	*Sa*	35	~0.001	[0.00 – 0.32]	0.05	[0.00 – 0.44]
Juvenile survival	*Sj*	33	0.310	[0.00 – 0.81]	0.06	[0.00 – 0.27]
Age at sexual maturity	*Lα*	114	~0.001	[0.00 – 0.99]	0.35	[0.00 – 0.78]
Clutch size	*CS*	253	0.990	[0.97 – 0.99]	0.99	[0.97 – 0.99]
Mean number of clutches per year	*CN*	98	0.920	[0.77 – 0.97]	0.62	[0.35 – 0.79]
Maximum lifespan	*ML*	207	0.820	[0.65 – 0.91]	0.77	[0.70 – 0.81]

We also compare the phylogenetic signal of the residuals used in the *p*PCA in the manuscript. The residuals between each life history trait and the female body mass, log-transformed. The results support the use of the phylogenetic PCA due to the high phylogenetic signal of the residuals in most of the traits (Supplementary Table 8).

Supplementary Table 8. Phylogenetic signal of the residuals between life history traits and body mass in Testudines and Crocodilia. Pagel's λ describes the statistical dependence among species' trait values due to their phylogenetic relationships, ranging between 1 (meaning a life history trait is fully related to the phylogenetic structure as explained by Brownian motion), and 0 (meaning no phylogenetic structuring of the trait). Pagel's λ was calculated for all traits after the PGLS between trait and body mass. The variance for imputed data was calculated with the standard deviation of the 40 datasets

Life history trait	Symbol	Pagel's λ of residuals	
Adult survival	*Sa*	0.16	±0.20
Juvenile survival	*Sj*	0.18	±0.24
Age at sexual maturity	*Lα*	0.28	±0.21
Clutch size	*CS*	0.96	±0.01
Mean number of clutches per year	*CN*	0.67	±0.08
Maximum lifespan	*ML*	0.77	±0.03

Supplementary References

1. Legendre, P., & Legendre, L. (2012). Numerical ecology (3rd ed.). Amsterdam, the Netherlands: Elsevier Science.
2. Salguero-Gómez, R., et al. (2016). COMADRE: A global data base of animal demography. Journal of Animal Ecology, 85(2), 371–384.
3. DATLife Database. (2021). Max-Planck Institute for Demographic Research (Germany). Available at www.datlife.org (data downloaded on [25-02-2021]
4. Myhrvold, N. P., et al. (2015). An amniote life-history database to perform comparative analyses with birds, mammals, and reptiles: Ecological Archives E096-269. Ecology, 96(11), 3109-3109.
5. Allen, W. L., Street, S. E., & Capellini, I. (2017). Fast life history traits promote invasion success in amphibians and reptiles. Ecology Letters, 20(2), 222-230.
6. Pfaller, J. B., Chaloupka, M., Bolten, A. B., & Bjorndal, K. A. (2018). Phylogeny, biogeography and methodology: a meta-analytic perspective on heterogeneity in adult marine turtle survival rates. Scientific reports, 8(1), 1-10.
7. Reinke, B. A., et al. (2022). Diverse aging rates in ectothermic tetrapods provide insights for the evolution of aging and longevity. Science, 376(6600), 1459-1466.
8. Caswell, H. (2001). Matrix population models: Construction, analysis, and interpretation (2nd ed.). Sunderland, MA: Sinauer Associates.
9. Salguero-Gómez, R., & Plotkin, J. B. (2010). Matrix dimensions bias demographic inferences: implications for comparative plant demography. The American Naturalist, 176(6), 710–722.
10. Van Buuren, S., & Groothuis-Oudshoorn, K. (2011). *mice*: Multivariate imputation by chained equations in R. Journal of statistical software, 45(1), 1-67.
11. Oksanen, J., et al. (2013). Package 'vegan'. Community ecology package, version, 2(9), 1-295.
12. Gaillard, J. M., et al. (1989). An analysis of demographic tactics in birds and mammals. Oikos, 59-76.
13. Healy, K., et al. (2019). Animal life history is shaped by the pace of life and the distribution of age-specific mortality and reproduction. Nature ecology & evolution, 3(8), 1217-1224.
14. Fraxe Neto, H. J., et al. (2011). Demography of *Acanthochelys spixii* (Testudines, Chelidae) in the Brazilian Cerrado. Chelonian Conservation and Biology, 10(1), 82-90.
15. Colston, T. J., Kulkarni, P., Jetz, W., & Pyron, R. A. (2020). Phylogenetic and spatial distribution of evolutionary diversification, isolation, and threat in turtles and crocodilians (non-avian archosauromorphs). BMC Evolutionary Biology, 20(1), 1-16.
16. Regis, K. W., & Meik, J. M. (2017). Allometry of sexual size dimorphism in turtles: a comparison of mass and length data. PeerJ, 5, e2914.
17. Cunha, F. A., Fernandes, T., Franco, J., & Vogt, R. C. (2019). Reproductive biology and hatchling morphology of the Amazon toad-headed turtle (*Mesoclemmys raniceps*)(Testudines: Chelidae), with notes on species morphology and taxonomy of the Mesoclemmys group. Chelonian Conservation

and Biology: Celebrating 25 Years as the World's Turtle and Tortoise Journal, 18(2), 195-209.

18. Fielder, D. P., Limpus, D. J., & Limpus, C. J. 2015. Reproduction and population ecology of the vulnerable western sawshelled turtle, *Myuchelys bellii*, in the Murray–Darling Basin, Australia. Australian Journal of Zoology, 62(6), 463-476.
19. Encyclopedia of Life. Available from http://eol.org. Accessed 15 September 2021
20. Andersen, S. K., et al. (2021). Economics, life history and international trade data for seven turtle species in Indonesian and Malaysian farms. Data in Brief, 34, 106708.
21. McCormack, T, et al. (2014). *Mauremys annamensis* (Siebenrock 1903)—Vietnamese Pond Turtle, Annam Pond Turtle, Rùa Trung Bộ. Conservation Biology of Freshwater Turtles and Tortoises.
22. Butterfield, T. G., Scoville, A., García, A., & Beck, D. D. (2018). Habitat use and activity patterns of a terrestrial turtle (*Rhinoclemmys rubida perixantha*) in a seasonally dry tropical forest. Herpetologica, 74(3), 226-235.
23. Lin, L., et al. (2018). Reproductive ecology of the endangered Beal's-eyed turtle, *Sacalia bealei*. PeerJ, 6, e4997.
24. López-Luna, M. A., et al. (2018). A distinctive new species of mud turtle from Western Mexico. Chelonian Conservation and Biology, 17(1), 2-13.
25. Rawski, M., & Józefiak, D. (2014). Body condition scoring and obesity in captive African side-neck turtles (Pelomedusidae). Annals of Animal Science, 14(3), 573-584.
26. Akani, G. C., Petrozzi, F., Segniagbeto, G. H., & Luiselli, L. (2015). Notes on morphology, biology and domestic consumption of *Pelusios niger* (Duméril & Bibron, 1835) from Forcados River, Nigeria. HERPETOZOA, 28(1-2), 94-98.
27. Chiari, Y., et al. (2017). Self-righting potential and the evolution of shell shape in Galápagos tortoises. Scientific reports, 7(1), 1-8.
28. McDonald, N. 2008. *Gopherus flavomarginatus*. Mexican Gopher Tortoise. https://animaldiversity.org/accounts/Gopherus_flavomarginatus/
29. Hailey, A., & Coulson, I. M. 1996. Temperature and the tropical tortoise *Kinixys spekii*: constraints on activity level and body temperature. Journal of Zoology, 240(3), 523-536.
30. Bonin, F., Devaux, B., & Dupré, A. (2006). Turtles of the World. JHU Press.
31. Gramentz, D. (2008). African Flapshell Turtles: The Genera Cyclanorbis and Cycloderma. Ed. Chimaira.
32. Freckleton, R. P. (2000). Phylogenetic tests of ecological and evolutionary hypotheses: Checking for phylogenetic independence. Functional Ecology, 14(1), 129–134.
33. Revell, L. J. (2012). *phytools*: An R package for phylogenetic comparative biology (and other things). Methods Ecol. Evol., 3(2), 217–223.
