# Supplementary material for: Anthropogenic impacts on threatened species erode functional diversity in chelonians and crocodilians
Source: Nat Commun. 2023 Mar 28;14:1542. doi: 10.1038/s41467-023-37089-5 (PMC10050202; doi:10.1038/s41467-023-37089-5)
Supplement: Supplementary file 2 — Description of Additional Supplementary Files [file 41467_2023_37089_MOESM2_ESM.pdf]

Description of Additional Supplementary Files

File Name: Supplementary Data 1

Description: Data with the life history traits of the 236 species of Testudines and 23 species of Crocodilia examined in this study. The life history traits are: adult survival (Sa), juvenile survival (Sj), maximum lifespan (ML), age at sexual maturity (Lα), mean of number of clutches per year (CN), clutch size (CS).

File Name: Supplementary Data 2

Description: Excel file with the Supplementary Table 2. Scores of phylogenetically-corrected principal component analysis (pPCA), also corrected by body mass, for the 236 species of Testudines and 23 species of Crocodilia examined in this study

File Name: Supplementary Data 3

Description: Excel file with the Supplementary Table 5. List of species used in the manuscript for which females body mass (g.) was available.

File Name: Supplementary Data 4

Description: Data with the threats of the 236 species of Testudines and 23 species of Crocodilia examined in this study. Threats are divided in i) habitat loss, fragmentation, and degradation, ii) over-collection of individuals and their eggs for food consumption, iii) unsustainable or illegal international trade, as well as over-collection for the trade in medicines, iv) climate change; v) interaction with invasive species and diseases.

File Name: Supplementary Software

Description: Zip file with all the code and data to run the models of the manuscript. Detailed information is included in “Readme.txt”
