# Supplementary material for: Anthropogenic impacts on threatened species erode functional diversity in chelonians and crocodilians
Source: Nat Commun. 2023 Mar 28;14:1542. doi: 10.1038/s41467-023-37089-5 (PMC10050202; doi:10.1038/s41467-023-37089-5)
Supplement: Supplementary file 8 — Reporting Summary [file 41467_2023_37089_MOESM8_ESM.pdf]

nature portfolio

Corresponding author(s): Roberto C. Rodríguez-Caro
Rob Salguero-Gómez

Last updated by author(s): 2022/02/09

# Reporting Summary

Nature Portfolio wishes to improve the reproducibility of the work that we publish. This form provides structure for consistency and transparency in reporting. For further information on Nature Portfolio policies, see our Editorial Policies and the Editorial Policy Checklist.

## Statistics

For all statistical analyses, confirm that the following items are present in the figure legend, table legend, main text, or Methods section.

| n/a | Confirmed | |
|---|---|---|
| ☐ | ☒ | The exact sample size ($n$) for each experimental group/condition, given as a discrete number and unit of measurement |
| ☐ | ☒ | A statement on whether measurements were taken from distinct samples or whether the same sample was measured repeatedly |
| ☐ | ☒ | The statistical test(s) used AND whether they are one- or two-sided<br>*Only common tests should be described solely by name; describe more complex techniques in the Methods section.* |
| ☐ | ☒ | A description of all covariates tested |
| ☐ | ☒ | A description of any assumptions or corrections, such as tests of normality and adjustment for multiple comparisons |
| ☐ | ☒ | A full description of the statistical parameters including central tendency (e.g. means) or other basic estimates (e.g. regression coefficient) AND variation (e.g. standard deviation) or associated estimates of uncertainty (e.g. confidence intervals) |
| ☐ | ☒ | For null hypothesis testing, the test statistic (e.g. $F$, $t$, $r$) with confidence intervals, effect sizes, degrees of freedom and $P$ value noted<br>*Give P values as exact values whenever suitable.* |
| ☒ | ☐ | For Bayesian analysis, information on the choice of priors and Markov chain Monte Carlo settings |
| ☒ | ☐ | For hierarchical and complex designs, identification of the appropriate level for tests and full reporting of outcomes |
| ☒ | ☐ | Estimates of effect sizes (e.g. Cohen's $d$, Pearson's $r$), indicating how they were calculated |

*Our web collection on statistics for biologists contains articles on many of the points above.*

## Software and code

Policy information about availability of computer code

| | |
|---|---|
| Data collection | No software was used for data collection. |
| Data analysis | All the analyses were done in R and the packages that we used were: phytools, mice, ks, mgcv and ggplot2. All the codes and complemetray packages are provided in the supplementary information. |

For manuscripts utilizing custom algorithms or software that are central to the research but not yet described in published literature, software must be made available to editors and reviewers. We strongly encourage code deposition in a community repository (e.g. GitHub). See the Nature Portfolio guidelines for submitting code & software for further information.

## Data

Policy information about availability of data

All manuscripts must include a data availability statement. This statement should provide the following information, where applicable:

- Accession codes, unique identifiers, or web links for publicly available datasets
- A description of any restrictions on data availability
- For clinical datasets or third party data, please ensure that the statement adheres to our policy

All data are available in the supplementary materials. Data was obtained from the COMADRE Animal Matrix Database [ref 62], DATLife Database [63], Amniote Life History Database [64], and the published reviews [65-67] . Phylogenetic tree from a previous published manuscript [21]

## Human research participants

Policy information about studies involving human research participants and Sex and Gender in Research.

| | |
|---|---|
| Reporting on sex and gender | NA |
| Population characteristics | NA |
| Recruitment | NA |
| Ethics oversight | NA |

Note that full information on the approval of the study protocol must also be provided in the manuscript.

# Field-specific reporting

Please select the one below that is the best fit for your research. If you are not sure, read the appropriate sections before making your selection.

☐ Life sciences ☐ Behavioural & social sciences ☒ Ecological, evolutionary & environmental sciences

For a reference copy of the document with all sections, see nature.com/documents/nr-reporting-summary-flat.pdf

# Ecological, evolutionary & environmental sciences study design

All studies must disclose on these points even when the disclosure is negative.

**Study description**

We described their life history strategies (i.e., schedules of survival, development, and reproduction) corrected by body mass and phylogeny using phyloPCA. We estimated the loss of functional diversity in simulated extinction scenarios using IUCN Red List by GAM models, we also evaluated these values using simulations of 1000 random null models. Finally we evaluated the relation of threats and the specific functional strategies using GAM models.

**Research sample**

We examined 259 species of Testudines and Crocodilia. We used the species when, at lest, one life history trait is available before the imputation and, also, we have phylogenetic information in tree [21]

**Sampling strategy**

We used all the species with information of life history traits and phylogeny which were avaliable.

**Data collection**

Data was collected by Roberto Rodríguez.
To calculate the life history strategies of Testudines and Crocodilia, we collected/estimated the life history traits according to the different datasets:
- Mean number of clutches per year and clutch size (CN and CS, respectively) were obtained from Amniote [64] and Allen et al. [65]. Allen et al. [65] includes several data (including Amniote) and, when they found multiple records of the same trait for a species, the average of the species' trait was estimated by taking the mean of unique records per species. When the same species was present in both datasets, we selected the value in Allen et al. [65].
- Adult and juvenile survival (Sa and Sj, respectively) were calculated from the MPMs in COMADRE[62], life tables in DATLife[63], and from direct estimates of capture-recapture published studies[66,67]. Adult survival (Sa) from MPMs was estimated as the column sum of the matrix U of the stages representing reproductive individuals represented in the sub-matrix F (considering adults, all ages/stages after the first reproduction). We used arithmetic averages for the resulting stage-specific survival values, rather than weighting them by the stable stage distribution because we collected juvenile and adult data from other sources where calculating this weighted mean would not feasible, thus rendering comparisons impossible. In DATLife, we used age-specific fertility to determine the reproductive ages, and then estimated adult survival as the arithmetic average of age-specific survival rates after the first reproduction. We estimated juvenile survival (Sj) using a similar approach: as the average of the sum of the columns that represent juveniles (all the stages prior to the first reproduction) in the pertinent sub-matrix U in COMADRE. In DATLife, we estimated the average of non-reproductive age- or stage-specific survival (prior to the first reproduction) of the life tables.
- Age at maturity (Lα) and maximum lifespan (ML) were estimated with the MPMs from COMADRE or obtained directly from the databases with this information available (such as Amniote or DATLife). In COMADRE, Lα was calculated using age-from-stage decompositions. Briefly, we defined the reproductive stages as those columns of the F sub-matrix that contain values greater than zero. We estimated the mean time between birth and the first entry into the reproductive stage. This conditional mean is obtained by creating an absorbing state corresponding to the event of reproducing at least once before death, with a Markov chain, and calculating the mean time to absorption. ML was estimated from the MPMs of COMADRE projecting 100 individuals in the first state and iterating up to 1000 years to identify the first year with fewer than one individual in the virtual cohort. We also used the databases Amniote and DATLife because they contain information about Lα and ML of most of the examined species. When several values from different datasets for one species and life history trait were available, we selected the lowest value for Lα and the highest values for ML. The rationale behind this choice is that the lowest value of Lα identifies the most likely first age at maturity reported in the life cycle of this species, whereas the highest values of ML approximate the maximum values of this trait reported for the species of interest in wildlife populations.
-We collected the information on the threats to each species from Stanford et al. [26] , Bonin et al. [92] and the IUCN Red List[23]. We did dichotomous variables when one of these threats were mentioned. We were not able to quantify the effect of the threat, because effect size is only available for the Critically Endangered species[26]. To convert the threat descriptions of the Red List into

| | |
|---|---|
| | our broader categories, we used the following process: for habitat degradation we considered the Red List threat classifications "residential & commercial development", "agriculture & aquaculture" and "natural system modifications"; for climate change the classification "climate change and extreme weather"; and for disease the classification "invasive and other problematic species, genes & diseases". |
| Timing and spatial scale | Data was collected from 01/06/2021 to 01/06/2022 |
| Data exclusions | No data were excluded from the analyses |
| Reproducibility | All the analyses can be reproducible with the provided data and code in supplementary material. |
| Randomization | We just used randomization to evaluate the null model of loss of functional diversity. We do it to evaluate if the number of loss species are affecting the values of loss of functional diversity. |
| Blinding | Blinding was not relevant because we use all the species with available information. |

Did the study involve field work? ☐ Yes ☒ No

# Reporting for specific materials, systems and methods

We require information from authors about some types of materials, experimental systems and methods used in many studies. Here, indicate whether each material, system or method listed is relevant to your study. If you are not sure if a list item applies to your research, read the appropriate section before selecting a response.

## Materials & experimental systems

| n/a | Involved in the study |
|---|---|
| ☒ | ☐ Antibodies |
| ☒ | ☐ Eukaryotic cell lines |
| ☒ | ☐ Palaeontology and archaeology |
| ☒ | ☐ Animals and other organisms |
| ☒ | ☐ Clinical data |
| ☒ | ☐ Dual use research of concern |

## Methods

| n/a | Involved in the study |
|---|---|
| ☒ | ☐ ChIP-seq |
| ☒ | ☐ Flow cytometry |
| ☒ | ☐ MRI-based neuroimaging |
